# HOXA5 Participates in Brown Adipose Tissue and Epaxial Skeletal Muscle Patterning and in Brown Adipocyte Differentiation

**DOI:** 10.3389/fcell.2021.632303

**Published:** 2021-02-25

**Authors:** Miriam A. Holzman, Abigail Ryckman, Tova M. Finkelstein, Kim Landry-Truchon, Kyra A. Schindler, Jenna M. Bergmann, Lucie Jeannotte, Jennifer H. Mansfield

**Affiliations:** ^1^Department of Biology, Barnard College, Columbia University, New York, NY, United States; ^2^Centre de Recherche sur le Cancer de l’Université Laval, CRCHU de Québec-Université Laval (Oncology), Québec City, QC, Canada

**Keywords:** *Hoxa5*, brown adipose tissue, adipose development, skeletal muscle development, differentiation

## Abstract

Brown adipose tissue (BAT) plays critical thermogenic, metabolic and endocrine roles in mammals, and aberrant BAT function is associated with metabolic disorders including obesity and diabetes. The major BAT depots are clustered at the neck and forelimb levels, and arise largely within the dermomyotome of somites, from a common progenitor with skeletal muscle. However, many aspects of BAT embryonic development are not well understood. *Hoxa5* patterns other tissues at the cervical and brachial levels, including skeletal, neural and respiratory structures. Here, we show that *Hoxa5* also positively regulates BAT development, while negatively regulating formation of epaxial skeletal muscle. HOXA5 protein is expressed in embryonic preadipocytes and adipocytes as early as embryonic day 12.5. *Hoxa5* null mutant embryos and rare, surviving adults show subtly reduced iBAT and sBAT formation, as well as aberrant marker expression, lower adipocyte density and altered lipid droplet morphology. Conversely, the epaxial muscles that arise from a common dermomyotome progenitor are expanded in *Hoxa5* mutants. Conditional deletion of *Hoxa5* with *Myf5/Cre* can reproduce both BAT and epaxial muscle phenotypes, indicating that HOXA5 is necessary within *Myf5-*positive cells for proper BAT and epaxial muscle development. However, recombinase-based lineage tracing shows that *Hoxa5* does not act cell-autonomously to repress skeletal muscle fate. Interestingly, *Hoxa5*-dependent regulation of adipose-associated transcripts is conserved in lung and diaphragm, suggesting a shared molecular role for *Hoxa5* in multiple tissues. Together, these findings establish a role for *Hoxa5* in embryonic BAT development.

## Introduction

Brown adipose tissue (BAT) mediates non-shivering thermogenesis in mammals. It is also an endocrine and immune organ that regulates metabolism and lipid storage both via endocrine and paracrine signaling, and through its own high metabolic activity (Jung et al., 2019). Understanding BAT development and physiology is critical for understanding human health, and has therapeutic applications for controlling obesity, diabetes, and other metabolic disorders. Despite exciting recent progress, however, our knowledge of BAT development is incomplete.

In mice and human infants, most BAT is clustered in depots at the forelimb and cervical levels, with smaller deposits found around organs such as the kidney and gonads. The major BAT depots are the interscapular BAT (iBAT), scapular (axillary) BAT (sBAT), and cervical BAT (cBAT) (Zhang et al., 2018; Jung et al., 2019). Brown adipocytes are distinguished by their abundant mitochondria located in proximity to stores of lipids in multilocular droplets that can be rapidly oxidized. These mitochondria express the inner membrane protein UCP1, which uncouples the proton gradient leak from ATP synthesis, generating heat. BAT is highly vascularized, permitting high oxygen levels necessary for its metabolic activity and facilitating rapid heat transfer through the circulation. This non-shivering form of thermogenesis is critical for temperature regulation in most mammals, including in human infants. Communication between BAT and white adipose tissue (WAT), in part through insulin signaling, has been linked to obesity in human adults, and WAT can undergo conversion to BAT under cold stress and adrenergic stimulation, a phenomenon known as browning (Srivastava and Veech, 2019). For these reasons, BAT has become a target of investigation for therapies to treat obesity, diabetes, and other metabolic syndromes.

Developmentally, most BAT arises from skeletal muscle progenitors within dermomyotome, via a competitive lineage switch (Sanchez-Gurmaches and Guertin, 2014a; Wang and Seale, 2016). Dermomyotome is a compartment of somites, segmented structures that form along the length of the body axis. Dermomyotome gives rise to most skeletal muscles, as well as dorsal dermis, smooth muscle and vasculature (Scaal and Christ, 2004). At some axial levels, dermomyotome also gives rise to BAT. Although it is unknown exactly which somites contribute to BAT, they are likely to include primarily those at the brachial and cervical levels where the major BAT depots form. Dermomyotomes can be further subdivided: dorsal and central dermomyotomes contain progenitors of epaxial muscles, which comprise the deep muscles of the back. The major BAT depots develop interleaved with epaxial muscles and probably also arise from dorsal and/or central dermomyotomes, described further below. Ventral dermomyotome is the source of the hypaxial muscles, which include the pre-vertebral and distal intercostal muscles, the ventral body wall and limb musculature, as well as the diaphragm. A core set of myogenic transcription factors, Myf5, Mrf4, MyoD, and Myogenin, governs the differentiation of all skeletal muscles, with overlapping but distinct roles and timing of activation in epaxial compared to hypaxial muscle progenitors (Buckingham and Rigby, 2014).

Genetic lineage tracing in mouse has shown that most BAT in the major depots is derived from cells expressing *Meox1, Pax3*, *Pax7*, *Myf5*, and *En1* (Atit et al., 2006; Seale et al., 2008; Lepper and Fan, 2010; Sanchez-Gurmaches and Guertin, 2014b). Cumulatively, this localizes BAT progenitors to the central and dorsal dermomyotome, where epaxial muscle progenitors are also found, as proposed previously (Sebo and Rodeheffer, 2019). However, the recombinase-based approaches used in these studies did not label all BAT cells, and there was some discrepancy between studies, leaving open the possibility for a heterogeneous origin. For example, while nearly all adipocytes from iBAT and sBAT are *Myf5*-positive, more than half of cBAT adipocytes are not (Sanchez-Gurmaches and Guertin, 2014b). Tamoxifen-inducible *Pax7* lineage labeling showed that BAT and skeletal muscle progenitors have separated by embryonic day (E) 11.5 (Lepper and Fan, 2010), but the time of their separation is not otherwise known. The earliest-expressed BAT specification factor known, the transcription factor EBF2, initiates in *Myf5*-expressing somite cells at E11.5 (Wang et al., 2014).

Further study is needed to characterize BAT specification *in vivo*. However, two interlinked genetic pathways are known to be involved in the process, and it is clear that BAT differentiation involves repression of both skeletal muscle and WAT differentiation programs. In the first pathway, EBF2 directly activates *Prdm16* transcription (Rajakumari et al., 2013), and PRDM16 is in turn a transcriptional co-activator for BAT specification genes, including *Pparg*, whose product is considered a master regulator of adipocyte fate (both BAT and WAT). EBF2 also confers specificity to PPARγ in activating brown adipocyte specific genes (Rajakumari et al., 2013). PRDM16 acts within co-repressor complexes to inhibit the expression of genes specific to both WAT and skeletal muscle. Loss of either *Ebf2* or *Prdm16* leads to ectopic myoblast specification in primary BAT cultures and in cell lines; conversely, overexpression of either factor is sufficient to transform prospective myoblasts into adipocytes (Seale et al., 2008; Wang et al., 2014). These data indicate that skeletal muscle and BAT are alternative fates for a common dermomyotome progenitor. *In vivo*, however, neither *Ebf2* nor *Prdm16* loss of function mutants show ectopic muscle. Rather, in *Prdm16* mutants, BAT develops normally, despite ectopically expressing myogenic transcripts, and also shows a partial transformation toward WAT; WAT transformation is enhanced by double knockout of *Prdm16* and *Prdm3* (Seale et al., 2008; Harms et al., 2014). Similarly, *Ebf2* mutants show both transcriptional and cell morphological changes that indicate a transformation of embryonic BAT toward WAT (Rajakumari et al., 2013; Wang et al., 2014).

In the second known pathway, BMP7 expression, activated by EWS and YBX1, is necessary for BAT development, and both *Bmp7* and *Ews* loss-of-function neonates show a reduction in iBAT mass (Tseng et al., 2008; Park et al., 2013). BAT from *Bmp7* null mutants also presents modestly reduced mRNA levels for differentiated BAT markers such as *Prdm16* (Tseng et al., 2008). In *Ews* null mutants, brown adipocyte differentiation is severely impaired, with reduced lipid droplet formation and decreased protein expression of several BAT regulators including PRDM16, PPARγ and UCP1, (Park et al., 2013). BAT from *Ews* or *Bmp7* mutant embryos also show ectopic expression of myogenic transcription factors including MYH3, as do primary cells depleted of either *Ews1* or *Bmp7* (Park et al., 2013). At least one downstream target of BMP7 signaling in embryonic BAT is the SUMOylation factor SENP2, which post-translationally modifies proteins involved in BAT differentiation (Liang et al., 2019). Together, these findings place *Ews* and *Bmp7* upstream of *Ebf2* and *Prdm16*, but the phenotypic differences between these groups of genes indicate some distinct roles. No single mutations are known that completely abolish the embryonic development of BAT, likely due to extensive functional redundancy and pleiotropy of its key regulators.

The notion that BAT and skeletal muscle are alternative fates is further supported by findings that BAT area is expanded following deletion of positive regulators of myogenesis, including in *Pax7* mutant embryos and in *MyoD/Igf2* double mutants, where it is accompanied by de-repression of *Prdm16* (Borensztein et al., 2012; An et al., 2017). In addition, *MyoD/Myf5* double mutants lack all skeletal muscle, and have adipose tissue in place of epaxial, intercostal, and ventral body wall muscles (Kablar et al., 2003). Finally, under wild-type conditions, BAT pre-adipocytes initiate but then extinguish transcription of core myogenic factors *Myf5*, *MyoD* and *myogenin*, although they never express *Mrf4* (Timmons et al., 2007; Wang et al., 2014; Mayeuf-Louchart et al., 2019). While the timing of these events is not fully described, myogenic transcription was found to extinguish by E12.5-13.5 in sorted PDGFRα+, MYF5+ somite cells (Wang et al., 2014), and to become significantly downregulated between E14.5–E15.5 in dissected wild-type iBAT (Mayeuf-Louchart et al., 2019).

Given that BAT depots arise at restricted axial positions, *Hox* genes emerge as excellent candidates to participate in their developmental origin. *Hoxa5* is part of the expression signature of adult brown adipocytes (Wang et al., 2014), and it promotes differentiation of brown preadipocytes in adult primary cultures (Cao et al., 2018b) as well as during white adipocyte browning (Cao et al., 2018a). However, the roles of *Hox* genes in adipose tissue development have not been addressed.

The HOXA5 protein expression domain extends from the third cervical through the second thoracic segments (C3-T2), which includes the somites located at the same axial level as the major BAT depots. Indeed, Cre-mediated fate mapping revealed that *Hoxa5* expressing cells contribute extensively to BAT including preadipocytes, adipocytes and connective tissue fibroblasts (Holzman et al., 2018). Together, these findings raise the question of whether *Hoxa5* regulates embryonic BAT development.

Here, we show that HOXA5 protein is expressed in embryonic, fetal and postnatal BAT. *Hoxa5* loss of function perturbs some features of BAT development, leading to a subtle reduction in BAT formation including in both the iBAT and sBAT depots, and reduction in adipocyte density and lipid droplet formation. Conversely, HOXA5 appears to negatively regulate the development of epaxial musculature that shares an embryonic origin with BAT. However, *Hoxa5* is not necessary for the lineage switch between muscle and brown adipocytes, as shown by fate mapping *Hoxa5*-expressing cells in a *Hoxa5* null background. The phenotypic effects of *Hoxa5* are apparent as early as E13.5, based on gene expression analysis. Both overall BAT reduction and epaxial muscle expansion can be recapitulated by the conditional deletion of *Hoxa5* in the *Myf5* lineage.

## Materials and Methods

### Mouse Lines and Breeding

The following mouse alleles were used: *Hoxa5*^–^ null allele: *Hoxa5*^tm 1Rob^ (Jeannotte et al., 1993); *Hoxa5*^flox^ conditional allele; *Hoxa5*^tm 1.1Ljea^ (Tabariès et al., 2007); *Hoxa5/Cre-GFP: Tg*^(14.5kb Hoxa5–*cre*)*Ljea*^ (Bérubé-Simard and Jeannotte, 2014); *Rosa26*^nYFP^*Cre* reporter: *B6.129* × *1 Gt(ROSA)26Sortm1(EYFP)Cos/J* (Srinivas et al., 2001); *Rosa26*^tdTomat^*^o^ Cre reporter:B6.Cg-Gt(ROSA)26Sortm9(CAG-tdTomato)Hze/J* (Madisen et al., 2010), *Meox1*^Cre:^ *Meox1*^tm 1(cre)Jpa^ (Jukkola et al., 2005); *Myf5*^Cre^: B6.129S4-*Myf5*^tm 3(cre)Sor^/J (Tallquist et al., 2000).

To generate embryos, animals of the appropriate genotype were crossed. Embryonic age was assigned as 0.5 day on the morning of detection of a vaginal plug. In all cases, the *Cre* allele was crossed in from the male germline. For lineage mapping in the *Hoxa5* null background, the *Hoxa5*^–^ allele was recombined to the *Rosa26*^nYFP^ and *Rosa26*^tdTomato^ reporter chromosomes. To generate embryos, females bearing a *Hoxa5^–^, Rosa26*^nYFP^ or *Hoxa5^–^,Rosa26*^tdTomat^*^o^* chromosome were crossed to *Tg*^Hoxa5/Cre^*; Hoxa5*^–/+^ males.

All procedures were performed in accordance with the NIH Guide for Care and Use of Laboratory Animals and approved by the Columbia University IACUC.

### Genotyping

Genotyping was performed on tail biopsies or embryonic yolk sacs using GoTaq PCR mix (Promega) following manufacturer’s instructions. Primer sequences used are (from 5′ to 3′): Cre: forward GCGGTCTGGCAGTAAAAACTATC, reverse GTGAA ACAGCATTGCTGTCACTT; tdTOMATO/RFP: forward CTG TTCCTGTACGGCATGG, reverse GGCATTAAAGCAGCGTA TCC; YFP/GFP: forward GCACGACTTCTTCAAGTCCG CCATGCC, reverse GCGGATCTTGAAGTTCACCTTGATGCC; *Hoxa5*^+^ allele: forward ACTGGGAGGGCAGTGCCCC CACTTAGGACA, reverse CTGCCGCGGCCATACTCATGCT TTTCAGCT; *Hoxa5*^–^ allele forward ACTGGGAGGG CAGTGCCCCCACTTA GGACA, reverse GGCTACCT- GCCCATTCGACCACCAAGCGAA; *Hoxa5*^fl^ allele forward, CAGCAGCGATCTGCATTCAC, reverse GAAACGCACTGAAGCACTAC. The *Tg*^Hoxa5/Cre^ allele interferes with distinguishing between *Hoxa5*^–^ null heterozygotes and homozygotes because it gives a positive signal for the wild-type *Hoxa5*^+^ allele. There are no sequences unique to the *Hoxa5*^+^ allele and absent from both the *Hoxa5*^–^ and *Tg*^Hoxa5/Cr^*^e^* alleles that could be used for DNA genotyping, so we distinguished *Tg*^Hoxa5/Cre^; *Hoxa5*^–/–^ homozygotes from *Tg*^Hoxa5/Cre^; *Hoxa5*^+/–^ heterozygotes by extracting total RNA and performing qRT-PCR with *Hoxa5* primers as described above.

### BAT Measurements

For embryonic BAT depot measurements, whole body weight was measured immediately following embryo collection, and each depot was dissected dry, without buffer, and placed into a pre-weighed aluminum dish. Depots were dried completely and weighed on a Mettler-Toledo XL6 microbalance [fresh dissected tissue could not be stably weighed due to continuous evaporation from such small samples; dry weights ranged from ∼0.3 mg (smallest cBAT depot) to 3 mg (largest iBAT depot)]. For adult BAT measurements, fresh BAT depot weight was measured immediately following dry dissection without buffer.

Cross-sectioned BAT was photographed under pseudo-darkfield conditions in which it is reflective and easy to identify (identity was confirmed by labeling with PPARγ, not shown). BAT area was measured in light micrographs on 1 section per slide for 7 consecutive slides per embryo spanning the C6-T1 region (each slide contained 10 × 20 μm sections). To ensure that the exact same axial positions were compared in experimental vs. littermate control embryos, multiple sections and slides spanning the C6-T2 region were visualized, and both dorsal and ventral morphological landmarks were used to match section axial levels. Next, for each of the 7 tissue sections chosen per embryo, cumulative BAT area (iBAT, sBAT, and cBAT) was measured by drawing an outline in Photoshop and measuring the enclosed area. The resulting area measurements were added together, and the sum area over 7 sections (7 slides) compared between littermate controls. Paired t-tests were conducted on 3 littermate pairs using Prism 8.0.

### Cell Density Measurements

iBAT was stained with DAPI (and PPARγ to confirm adipocyte identity, not shown). Six littermate pairs (from 5 independent litters) of *Hoxa5*^–/–^ and *Hoxa5*^+/+^ were tested. For each embryo, the number of adipocytes was counted in 4 independent 100 μm^2^ fields of view and averaged.

### Immunofluorescence

**FIGURE 1:**
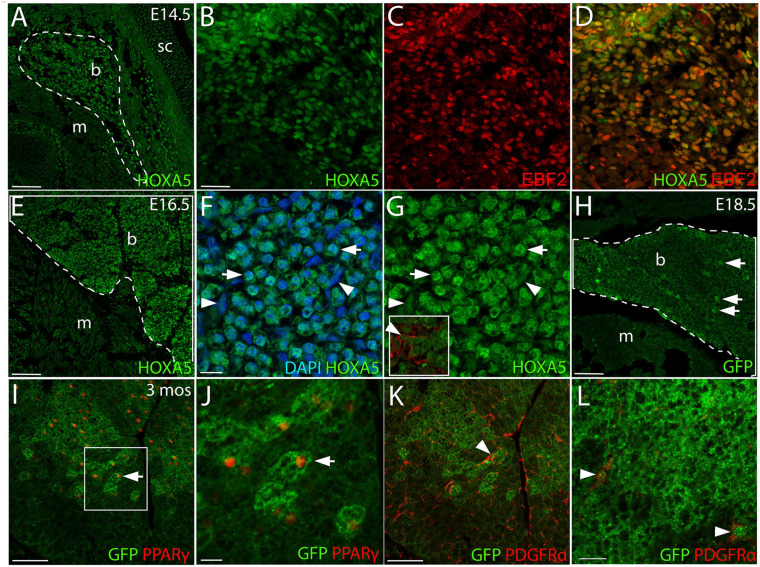
HOXA5 expression in embryonic and adult BAT. **(A)** HOXA5 is expressed throughout BAT from the first stage it becomes morphologically identifiable at E14.5 (white outline). **(B–D)** BAT adipocyte nuclei, identified based on expression of EBF2, co-express HOXA5. **(E)** HOXA5 continues to be expressed in BAT (white outline) at E16.5, shown here in sBAT. **(F,G)** HOXA5 is apparent in nuclei of round adipocytes (arrows) and sickle-shaped fibroblasts (arrowheads) within BAT. Inset in **(G)** shows that BAT fibroblasts are co-labeled with HOXA5 and PDGFRα. **(H)** By E18.5, cytoplasmic GFP reveals expression of *Hoxa5* in adipocytes (arrows) of a *Tg*^Hoxa5Cre/GFP^ embryo, also shown in sBAT. **(I,J)** At 3-months of age, *Hoxa5* expression in BAT remains detectable by GFP accumulation in a *Tg*^Hoxa5Cre/GFP^ adult mouse, and is mostly restricted to adipocytes that co-labeled with PPARγ- (arrows). Panel **(J)** is an inset of **(I)**, as indicated. **(K,L)** *Hoxa5* is also expressed in PDGFRα-positive fibroblasts (arrowheads). b, BAT; m, epaxial skeletal muscle; sc, scapula. Scale bars: 100 μm **(A,E,I,K)**, 50 μm **(B–D)**, 10 μm **(F,G)**, 200 μm **(H)**, 20 μm **(J,L)**. In all images, dorsal is up and lateral is to the right.

**FIGURE 2:**
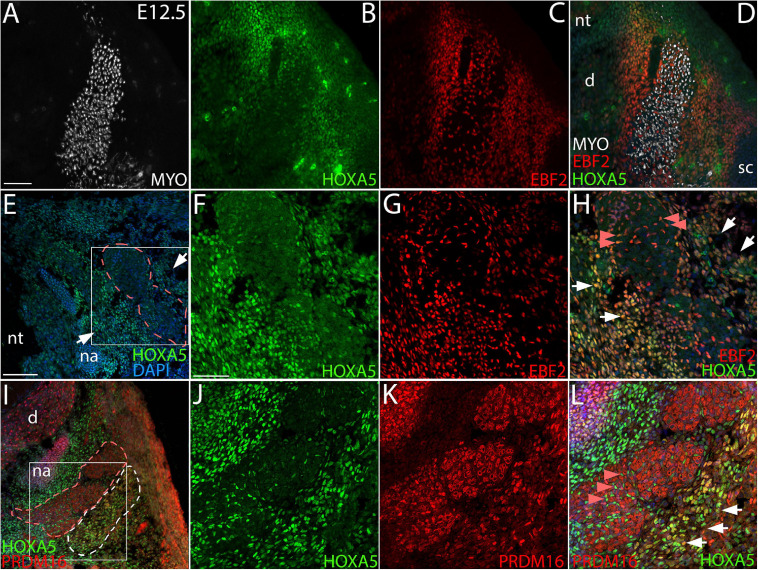
Co-localization of HOXA5 with BAT markers EBF2 and PRDM16 at E12.5. **(A–D)** The myotome, labeled with Myosin 4 (MYO) **(A)**, is flanked by mesenchyme that co-expresses HOXA5 and EBF2 **(B–D)**, and extends laterally to the scapula and medially to the neural tube. Panels **(E-L)** show higher magnification views of the area surrounding myotome; indicated insets in **(E,I)** are shown in **(F–H,J–L)**. **(E–H)** Epaxial myotome (dotted red outline in **(E)** is flanked by mesenchyme co-expressing HOXA5 and EBF2 (white arrows). While neither protein is expressed in muscle, co-expression is observed in prospective muscle connective tissue fibroblasts within the myotome (red arrowheads). **(I–L)** PRDM16 and HOXA5 are co-expressed in mesenchyme lateral to the epaxial myotome. The epaxial myotome is surrounded by a dotted red outline, and area of nuclear PRDM16 expression by the dotted white outline in **(I)**. Co-expression of nuclear HOXA5 in PRDM16 domain is marked by white arrows **(L)**. Note in contrast to EBF2 and HOXA5, PRDM16 is not expressed in muscle connective tissue fibroblasts (red arrowheads). However, cytoplasmic PRDM16 is expressed in skeletal muscle. d, dorsal root ganglion, na, neural arch; nt, neural tube; sc, scapula. Scale bars: 200 μm **(A–D)**, 100 μm **(E,I)**, 50 μm **(F–H,J–L)**. In all images, dorsal is up and lateral is to the left.

**FIGURE 3:**
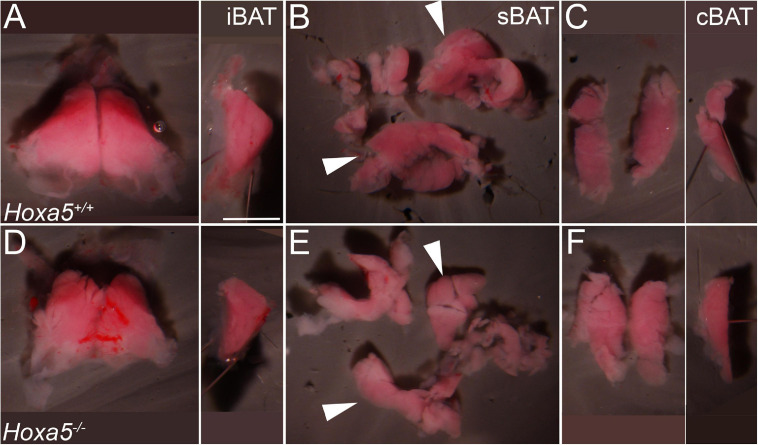
iBAT and sBAT reduction in E18.5 *Hoxa5* homozygous mutant embryos. BAT depots were dissected from *Hoxa5*^+/+^ **(A–C)** and *Hoxa5*^–/–^ **(D–F)** littermates. **(A,D)** iBAT was reduced but otherwise morphologically similar in a *Hoxa5* null embryo compared to the control. This is evident in ventral view (left) or in a medial view of one lobe (right image; anterior is up and dorsal is to the left). **(B,E)** sBAT was also reduced, particularly in the thickest part (white arrowheads) located just medial to the scapular blades. **(C,F)** cBAT appears similar in both genotypes, in frontal (left) or medial (right) views. iBAT, interscapular BAT; sBAT, scapular BAT, cBAT, cervical BAT. Scale bar: 2 mm.

**FIGURE 4:**
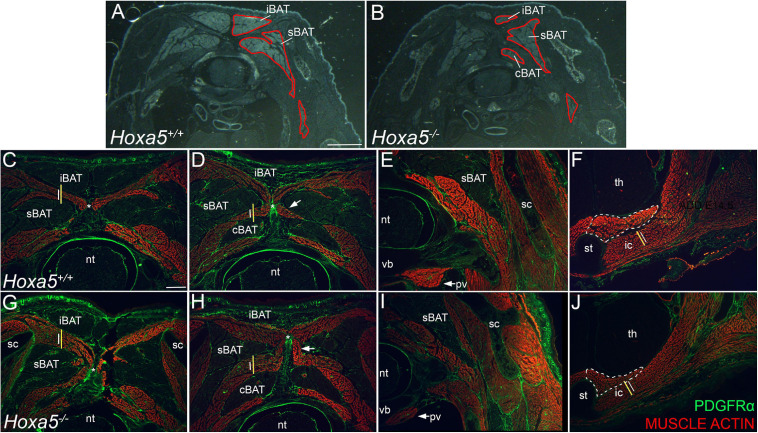
BAT reduction and epaxial skeletal muscle expansion in *Hoxa5* null embryos at E18.5. **(A,B)** Pseudo-darkfield micrographs through position-matched C7 segments show reduced BAT area in a *Hoxa5* null embryo compared to a littermate control. Depots are outlined in red on the right side only. **(C–J)** Epaxial muscles are expanded concomitant with BAT reduction, while some hypaxial muscles are reduced in *Hoxa5* null embryos. Skeletal muscles are stained with actin, and PDGFRα counterstains connective tissue fibroblasts within muscle and BAT, tendon, ligament, dermis. Wild type vs. *Hoxa5* null littermates were compared in position-matched sections through C7 and T1 segments. Lines reproduced in **(C,G)** compare the rhomboid height in wild-type (white) vs. *Hoxa5* null (yellow) at the same position. Similar lines in **(D,H)** compare relative splenius height. Both are larger in *Hoxa5* null embryos. The nuchal ligament is marked by an asterisk in **(C,D,G,H)**. Hypaxial muscles show reduced size, including the prevertebral muscle (arrows, **E,I**), the sternothyroid muscle (white dotted outline, **F,J**) and first intercostal muscle [white lines in **F**,**J** are reproduced at the same size to compare wild-type (white) and *Hoxa5* null (yellow) littermates]. cBAT, cervical BAT; iBAT, interscapular BAT; ic, intercostal muscle; nt, neural tube; pv, prevertebral muscle; sBAT, scapular BAT; sc, scapula; st, sternum; th, thymus; vb, vertebral body. Scale bars: **(A,F)** 2 mm; all other panels: 400 μm.

**FIGURE 5:**
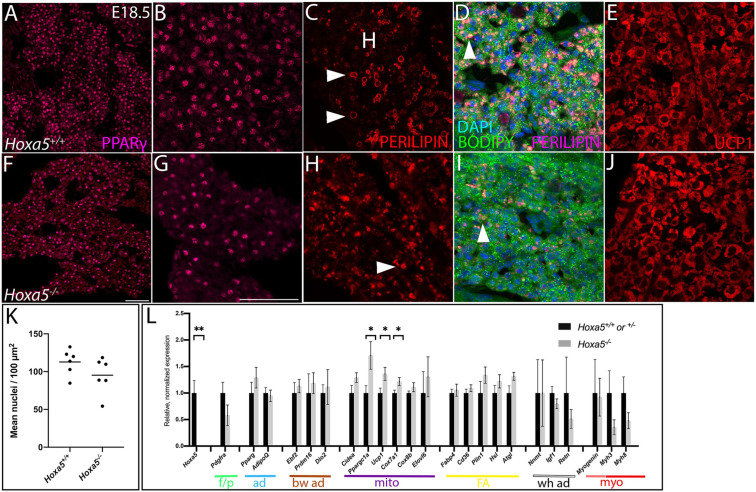
Cellular phenotypes of *Hoxa5* mutant BAT at E18.5. Nuclear adipocyte marker PPARγ **(A,B,F,G)** shows adipocytes are less dense in *Hoxa5* null BAT compared to littermate controls. **(C,D,H,I)** Lipid droplets (white arrowheads) visualized by Perilipin, which accumulation at their peripheries and/or the fluorescent lipid dye BODIPY are reduced in number and disorganized in *Hoxa5* null compared to control iBAT. BAT-specific inner mitochondrial membrane protein UCP1 showed similar accumulation in *Hoxa5* null compared to control BAT **(E,J)**. **(K)** Mean iBAT nuclear density indicates a trend toward reduction in *Hoxa5* null embryos (*n* = 6 littermate pairs). **(L)** qRT-PCR on dissected E18.5 sBAT shows expression of key markers in *Hoxa5* mutants. Color coded lines and abbreviations indicate transcript markers for specific cell types, including markers for fibroblasts/pre-adipocytes (p/f), transcriptional markers for all adipocytes (ad), transcriptional markers of brown adipocytes (bw ad), transcripts associated with mitochondrial biogenesis and thermogenic function (mito), transcripts associated with fatty acid uptake and storage (FA), with white adipocytes (wh ad), myogenic precursors (pink), differentiated muscle (red) (myo). All markers were quantified relative to the *Rpl19* control and normalized to the average value from control embryos. Mean and SEM of *n* = 5–6 embryo pairs shown. ^∗^*p* < 0.05, ^∗∗^*p* < 0.01, two-tailed *t*-test. Scale bars: 50 μm. (**A,F** are at the same scale; all other panels are at the same scale shown in **F**).

**FIGURE 6:**
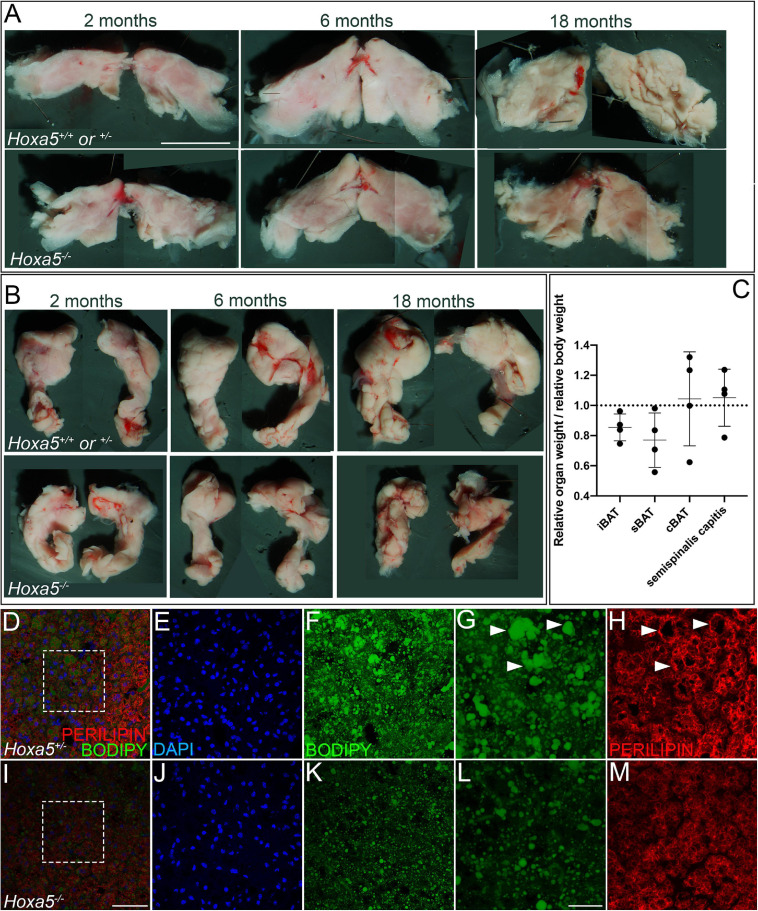
*Hoxa5* null BAT phenotypes persist in adults. Rare *Hoxa5^–/–^* animals that survived embryonic lethality were compared to *Hoxa5*^+^*^/^*^+^or ^±^ littermate controls. **(A,B)** iBAT or sBAT is reduced in *Hoxa5* null adults across a range of ages. iBAT is shown in ventral view **(A)** and sBAT in dorsal view **(B)**, with anterior up. **(C)** Relative weights of these organs in *Hoxa5^–/–^* animals compared to controls, normalized for whole-animal body weight, also showed reduction for both iBAT and sBAT. Measurements were pooled across 4 littermate controls of various ages. See also Supplementary Table 3. **(D–M)** sBAT sections were compared in a 2-month adult littermate pair; insets indicated in **(D,I)** are shown in **(E,F,J,K)**. BAT cell density visualized by DAPI **(E,J)** appeared reduced in *Hoxa5^–/–^* animals, similar to what was observed in embryos. In addition, lipid droplets were smaller and less organized in null compared to control sBAT, visualized with the lipophilic dye BODIPY (**F,G** vs. **K,L**) or with PERILIPIN IF **(H,M)**. Arrowheads in **(G,H)** indicate large, well defined lipid droplets stained by BODIPY and outlined by PERILIPIN that are common in wild-type but not in *Hoxa5* null iBAT. Scale bars: panels **(A,B)** 2 mm; **(D–F,I–K)** 50 μm; **(G,H,L,M)** 20 μm.

**FIGURE 7:**
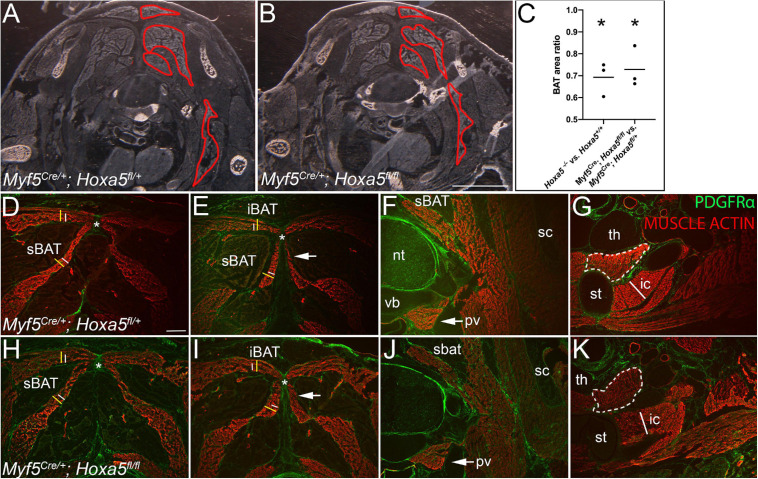
*Hoxa5* deletion in the *Myf5* lineage recapitulates BAT reduction and epaxial muscle expansion of *Hoxa5* null embryos. Control heterozygous **(A,D–G)** and conditionally *Hoxa5* deleted **(B,H–K)** E18.5 embryos of the genotypes indicated were examined for BAT and skeletal muscle phenotypes **(A,B)** Pseudo-darkfield micrographs through position-matched segments show reduced BAT area in *Hoxa5* null embryo compared to littermate control. Depots are outlined in red on the right side only. **(C)** Measurement of BAT area on tissue sections, as described in the text, shows it is significantly reduced following either complete deletion or *Myf5* conditional deletion of *Hoxa5.* Lines show mean BAT ratio of 69% for *Hoxa5* null embryos compared to controls, and 73% for embryos in which *Hoxa5* is conditionally deleted with *Myf5/Cre.* Asterisk indicates *p* < 0.05, paired *t*-test; *n* = 3 littermate pairs. **(B,C,G,H)** A reciprocal expansion of epaxial muscles was also observed. White and yellow lines duplicated in **(D,E)** and **(H,I)** compare epaxial muscle width in controls (white lines) vs. *Hoxa5* conditional deletion (yellow lines). The top lines mark the width of the rhomboid (note the trapezius overlying the rhomboid is not included). Bottom lines mark the splenius. The nuchal ligament is marked by an asterisk. **(F,G,J,K)** Hypaxial muscles show no difference following conditional *Hoxa5* deletion, including the prevertebral muscle (arrows, **F,J**), the sternothyroid muscle (white dotted outline, **G,K**) and first intercostal muscle (white line in **G,K** is reproduced at the same size in each panel). Abbreviations: cBAT, cervical BAT; iBAT, interscapular BAT; ic, intercostal muscle; nt, neural tube; pv, prevertebral muscle; sBAT, scapular BAT; sc, scapula; st, sternum; th, thymus; vb, vertebral body. Scale bars for **(A,F)** 2 mm; for all other panels, 400 μm.

**FIGURE 8:**
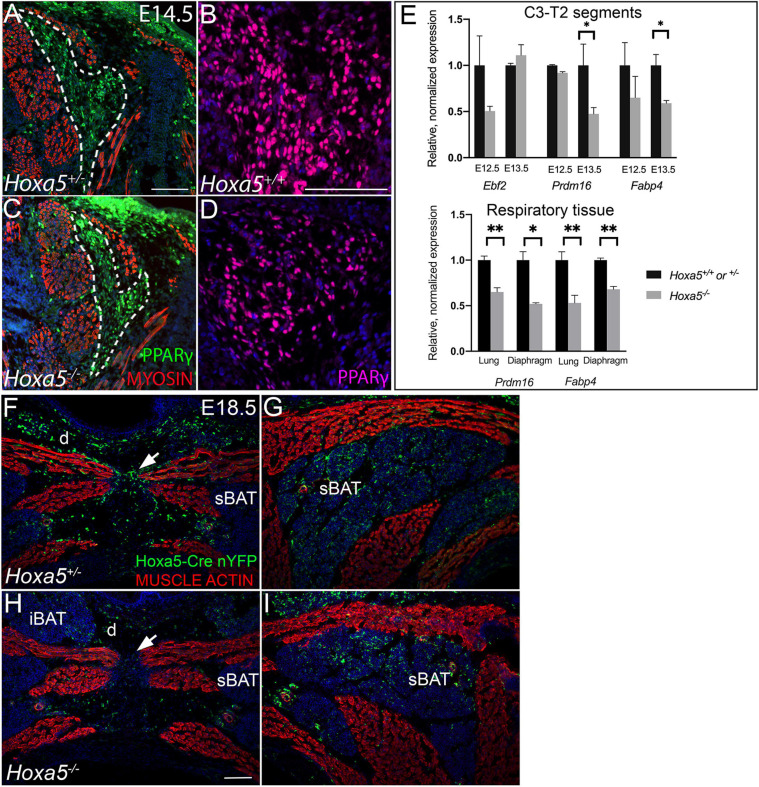
*Hoxa5* plays an early role in BAT development, but likely not in BAT versus skeletal muscle lineage specification. **(A–D)** *Hoxa5* null embryo shows smaller BAT area (dotted outline in **A,C**) and reduced adipocyte density (compare **B,D**) at E14.5, the earliest stage at which BAT is morphologically distinguished from skeletal muscle. **(E)** Dissected C3-T2 segments from E12.5–E13.5 *Hoxa5* null embryos at earlier stages show reduced expression of some early adipocyte transcripts, suggesting fewer specified BAT progenitors at this early stage. These transcripts were also reduced in *Hoxa5* null respiratory tissues, suggesting a conserved regulatory role for HOXA5 across tissues. Markers were quantified relative to the *rpl19* control and normalized to the average value from control embryos. Mean and SEM of *n* = 6 embryo pairs shown. ^∗^*p* < 0.05, ^∗∗^*p* < 0.01, two-tailed *t*-test. **(F–I)** *Hoxa5* -descendant cells show identical tissue-restriction in *Hoxa5* null embryos compared to controls, indicating *Hoxa5* is not necessary to cell-autonomously repress skeletal muscle fate. *Hoxa5* descendant cells (cells activating the *Hoxa5* promoter) were labeled with nuclear YFP (nYFP) to determine their tissue contribution in controls (*Tg*^Hoxa5Cre^*; Rosa26*^nYFP/+^; *Hoxa5*^±^, **F–G**) compared to *Hoxa5* null littermates (*Tg*^Hoxa5Cre^*; Rosa26*^nYFP/+^; *Hoxa5*^–/–^*;* **H–I**). In both genotypes, cells descended from *Hoxa5* expressing cells contributed substantially to all three BAT depots (labeled sBAT adipocytes indicated by arrows). Further, *Hoxa5* descendant cells contributed to most musculoskeletal tissue types except skeletal muscle, as previously reported. Labeled cells were found within muscle connective tissue fibroblasts (arrowheads), as previously reported for wild-type embryos. Together, this indicates *Hoxa5* is not necessary to cell-autonomously repress skeletal muscle fate. iBAT, interscapular BAT; sBAT, scapular BAT; cBAT, cervical BAT. Arrows indicate the nuchal ligament at the midline. Scale bars: **(A,C,F–I)** 200 μm, **(B,D)** 100 μm.

Immunostaining was performed following previously described protocols (Holzman et al., 2018; McGlinn et al., 2019). Briefly, embryos were fixed in 4% paraformaldehyde at 4°C and embedded in OCT. Sections of 6–20 μm were cut, dried at 37°C, and frozen at −80°C. After air drying and rinsing in PBS, sections were permeabilized and blocked in 0.3% Triton-X/5% normal serum/PBS for 1 h, except for antibodies marked otherwise in Supplementary Table 1 (^∗^ indicates permeabilization with citrate-based antigen retrieval (McGlinn et al., 2019) was required prior to block; ^∗∗^ indicates permeabilization by 10 min incubation in methanol at −20°C prior to block). Primary antibodies were diluted (see Supplementary Table 1) in blocking solution and incubated overnight at 4°C. After washing in PBS, slides were incubated 2–3 h at room temperature in whole IgG secondary antibodies conjugated to Cy5, Alexa Fluor 488, Alexa Fluor 594 or Alexa Fluor 647 (Jackson Immunoresearch) at 1:200 or 1:400. Slides were stained with DAPI and mounted in Prolong Diamond (Invitrogen). Confocal images for Figures 1, 2, 5, 6, 8 were captured on a Nikon A1 confocal unit attached to a XX inverted microscope. For all images to be compared to each other, all optical and confocal settings were held constant. In all cases, confocal laser power was set to below 1% and gain adjusted to avoid pixel saturation. Epifluorescence images for Figures 4, 7 were captured on a Nixon Eclipse E600FN with a Lumenera Infinity3 Camera, with all settings held constant across samples to be compared, and exposure set to avoid pixel saturation. Images for Figures 3, 5 were captured on a Nikon SMZ1500 dissecting microscope and Nikon Ds-Fi1 camera.

### Lipid Droplet Staining

Lipid droplets were stained by incubating slides for 30 min in 0.5 μg/mL BODIPY (Fisher D3922) in PBS after processing for immunofluorescence. Excess was removed by multiple washes in PBS.

### Hematoxylin and Eosin Staining

Staining was performed on OCT cryosections with an H & E kit (Abcam # ab245880) following manufacturer’s instructions. Slides were mounted in gelvatol (McGlinn et al., 2019).

### Quantitative Reverse Transcriptase-PCR (qRT-PCR)

For qRT-PCR on neck/trunk segments from the *Hoxa5* expression domain, C3-T2 segments were dissected from embryos of the appropriate ages, and forelimb tissue, neural tube, and thoracic organs were removed. RNA extraction was performed by Trizol method, cDNA synthesis was performed with the AMV first strand synthesis kit (Invitrogen), and qPCR with the Applied Biosystems Sybr Master I mix, all following manufacturer’s instructions. qRT-PCR was performed on a Roche Lightcycler 480 instrument. Primer sequences are given in Supplementary Table 2.

## Results

### Location of Embryonic BAT Depots in Mice

Identification of the anterior–posterior (AP) axial level at which BAT forms can facilitate identification of candidate regulatory genes. Supplementary Figure 1 shows the position of the three major depots relative to the vertebral column in E18.5 embryos. iBAT is located between C7-T5, cBAT extends from C1 to C7 and sBAT is located just beneath the scapular blades, with an AP axis position extending from approximately C3 to T2 on the dorsal side, but with thin, irregularly shaped tissue extending ventrally in both anterior and posterior directions. Thus, all three BAT depots lie primarily but not entirely within segments patterned by HOXA5 (C3-T2). However, it should be noted that the axial location of the somites that contribute to brown adipocytes has not been mapped.

### HOXA5 Protein Is Expressed in BAT Adipocytes and Fibroblasts

At E14.5, HOXA5 protein was highly expressed throughout BAT when the latter first becomes morphologically distinct from epaxial muscle (Figure 1A). HOXA5 adipocyte expression was confirmed by co-labeling for EBF2 (Figures 1B–D). At E16.5, HOXA5 expression continued to be high in BAT (Figures 1E–G) including both adipocytes and connective tissue fibroblasts. These data agree with our previous result that *Hoxa5*/Cre lineage tracing labels both PPARγ-positive brown adipocytes and PDGFRα-positive BAT fibroblasts (Holzman et al., 2018).

By E18.5, HOXA5 expression in BAT decreased and was not reliably detected by IF (not shown). However, the *Hoxa5*/Cre-GFP mouse line (Bérubé-Simard and Jeannotte, 2014), which contains an IRES-GFP coding sequence, allowed indirect detection of *Hoxa5* promoter activity. This revealed GFP signal in a subset of adipocytes (Figure 1H). Similarly, some adipocytes were labeled in BAT from 3-month old adults. Notably, while PPARγ was detected in only a subset of adipoctyes, virtually all of the GFP-positive adipocytes co-expressed PPARγ (Figures 1I,J). GFP expression was also detected in connective tissue fibroblasts of BAT, based on PDGDFRα co-expression (Figures 1K,L).

We next assessed HOXA5 expression in prospective brown adipocytes prior to the morphological emergence of BAT. The exact location of these cells is unknown, although lineage tracing has shown that they arise from dermomyotome. EBF2 and PRDM16 are the two earliest known regulators of brown adipocyte differentiation, and they initiate somitic expression at E11.5 and E12.5, respectively, in what are presumed to be adipocyte progenitors (Seale et al., 2008; Wang et al., 2014). We therefore compared HOXA5 expression to these markers. At E12.5, the epaxial muscle mass is flanked medially and laterally by mesenchyme, mostly somite derived, which will contribute to dermis, BAT, tendon and ligament. Triple labeling for epaxial muscle, EBF2 and HOXA5 (Figures 2A–D) showed that both HOXA5 and EBF2 are broadly co-expressed throughout this mesenchyme, extending laterally to the scapula and medially to the neural tube (Figures 2B–D). This broad spatial domain suggested that expression of both proteins includes but is not limited to prospective brown adipocytes. In closer view, neither protein is expressed in skeletal muscle (Figures 2E–H; red outline marks the epaxial muscle mass), consistent with our previous reports for HOXA5 (Landry-Truchon et al., 2017; Holzman et al., 2018). However, EBF2 and HOXA5 are co-expressed in muscle connective tissue (MCT) fibroblasts interspersed within the muscle (Figure 2H, red arrowheads). Their identity as MCT was based on their co-expression of HOXA5 with MCT markers PDGFRα and TCF4 (Holzman et al., 2018).

Nuclear PRDM16 is expressed in a more restricted spatial domain than EBF2, in mesenchyme lateral but not medial to the epaxial muscle mass. This domain of cells expressing nuclear PRDM16 co-expressed HOXA5 (Figures 2I–L). We hypothesize that this domain contains the prospective brown adipocytes. Unlike HOXA5 and EBF2, PRDM16 expression is absent from muscle connective tissue fibroblasts (Figures 2I–L). PRDM16 expression was detected in additional cell types, including dorsal root ganglia, cartilage, and in the cytoplasm (but not nuclei) of muscle cells (Figures 2J,K), consistent with previous reports that *Prdm16* mRNA is broadly expressed in all of these tissues (Lahortiga et al., 2004; Horn et al., 2011). Cytoplasmic PRDM16 protein localization in skeletal muscle we observed was not previously reported, although PRDM16 is known to shuttle between compartments (Pinheiro et al., 2012).

Together, the overlap of HOXA5 expression with EBF2 and nuclear PRDM16 in mesenchyme lateral to the epaxial muscle mass suggests that committed brown adipocytes most likely express HOXA5 at least from E12.5, and thus prior to the morphological emergence of BAT or the expression of key adipocyte differentiation factors such as PPARγ. However, further study is needed to identify a unique molecular signature of brown adipocytes and to definitively locate them during somite stages.

### *Hoxa5* Loss-of-Function Perturbs Fetal and Post-natal BAT and Axial Muscle Pattern

To test whether *Hoxa5* regulates BAT development, we next examined embryos homozygous for a *Hoxa5* targeted knockout that was previously shown to be a complete loss-of-function (null) allele [(Jeannotte et al., 1993) and reviewed in Jeannotte et al. (2016)]. The three major depots (iBAT, sBAT and cBAT) were dissected from E18.5 embryos (Figure 3). All were present and appeared morphologically similar in all genotypes. However, the size of iBAT and sBAT depots was visibly reduced in some *Hoxa5* null embryos compared to littermate controls (Figures 3A,B,D,E). The effect appeared most pronounced in the sBAT, the bulk of which is located directly beneath the scapular blade (Figures 3B,E, arrowheads and Supplementary Figure 1). Of 24 *Hoxa5^–/–^* vs. *Hoxa5*^+^*^/^*^+^ embryo littermate pairs (from 13 litters), the *Hoxa5* mutant sBAT appeared smaller than a paired wild-type or heterozygous control in 17 (71%), larger in 2 (8%), and approximately the same size in the remaining 5 pairs. iBAT appeared smaller in *Hoxa5* null mutant than in the paired littermate control in 12/27 pairs (44%), and larger in 5 (9%). In contrast, the cBAT did not appear affected in *Hoxa5* null embryos, or if anything was slightly larger in some samples (Figures 3C,F). To confirm this phenotype, BAT depots were dissected and depot dry weight measured (Supplementary Figure 2A). The buffer-free dissections produced substantial weight variation within genotypes, likely due to the contribution of small differences in transferred blood and connective tissue, and the small size of these structures (0.3–3 mg per depot). However, the same trend was observed, with significant overall reduction of sBAT (*p* = 0.024, one-tailed *t*-test). iBAT was not significantly different, and cBAT showed a small but significant increase in null embryos, similar to qualitative observations of area (*p* = 0.009, one-tailed *t*-test). The more anterior location of cBAT, and its differential origin mainly outside of the *Myf5* lineage, in contrast to sBAT and iBAT (Sanchez-Gurmaches and Guertin, 2014b), may be relevant to the differential effect of *Hoxa5* on these depots. Together, these data indicate a role for *Hoxa5* in BAT development, and in particular in positively regulating formation of the sBAT depot.

We next examined tissue sections for an *in situ* view, and a more quantitative assessment of the phenotype. Cumulative BAT area was measured in E18.5 embryos across a range of 7 position-matched sections spanning the C6-T1 segments in *Hoxa5^–/–^* to *Hoxa5*^+^*^/^*^+^ littermates. By this measurement, BAT area in *Hoxa5* mutant embryos was also significantly reduced to an average of 69% the area of paired littermate controls (Figures 4A,B; *p* = 0.015, paired one-tailed *t*-test, *n* = 3 embryo pairs). Together, the observations in whole BAT and in sections indicate that *Hoxa5* promotes sBAT and iBAT development, with a more pronounced effect on sBAT.

sBAT and iBAT reduction in *Hoxa5* null embryos coincided with an expansion in the area occupied by epaxial muscles, or deep muscles of the back, which develop interleaved between the BAT depots. Affected muscles included the rhomboid, splenius and semispinalis muscles, all of which appeared thickened in all *Hoxa5* null samples (Figures 4C,D,G,H). In contrast, several hypaxial muscles derived from the same somites were thinner, including the prevertebral (longus) muscles, the sternothyroid, and the intercostal muscles between T1–T2 (Figures 4E,F,I,J). This hypaxial phenotype was consistent with a previous report that *Hoxa5* null embryos have a reduced and thinner (hypaxial) diaphragm muscle with smaller myofibers (Landry-Truchon et al., 2017).

### Brown Adipocytes of *Hoxa5* Loss-of-Function Mutants Showed Reduced Density and Aberrant Lipid Droplet Morphology

We next assessed the cellular characteristics of sBAT and iBAT in *Hoxa5* mutant embryos. The cell density within both depots, and of PPARγ-positive adipocytes (the most abundant cell type in BAT), showed a trend toward decrease in *Hoxa5* null compared to wild-type embryos at E18.5 (Figures 5A,B,F,G,K). This difference was not significant (*p* = 0.08, one-tailed *t*-test, *n* = 6 littermate pairs). When pairwise comparisons were made between littermates, 2 showed significantly reduced adipocyte density (each *p* < 0.001) and 4 showed similar density in both genotypes, indicating that this, like many *Hoxa5* null phenotypes, was partially penetrant. This reduction was also observed by hematoxylin and eosin staining, which reveals all nuclei within the tissue (Supplementary Figures 2B–D).

We also examined *Hoxa5* null iBAT and sBAT for the two key specializations of differentiated brown adipocytes: presence of multilocular lipid droplets, and expression of UCP1, a protein that localizes to inner mitochondrial membranes and uncouples the proton gradient, producing heat. At E18.5, lipid droplets appeared disorganized and smaller in BAT from *Hoxa5* null mutants compared to controls. This was revealed by co-staining for Perilipin, which localizes to the perimeter of lipid droplets, and with the fluorescent dye BODIPY, which stains lipids within droplets (Figures 5C,D,H,I). We next examined lipid droplets in neonates, a stage when thermogenesis and BAT lipid metabolism is at a maximum (Hong et al., 2015). Due to embryonic lethality of *Hoxa5*, we generated a conditional knockout of *Hoxa5* in somites using the *Meox1*^Cre^ deleter mouse line (Jukkola et al., 2005). *Meox1-Cre* is expressed in all somites and derivatives as previously reported (Jukkola et al., 2005), including virtually all iBAT and sBAT adipocytes as well as skeletal muscle (not shown). As in embryos, lipid droplets in *Meox1*^Cre^; *Hoxa5*^fl/fl^ neonates were smaller and less organized relative to controls (Supplementary Figure 3).

UCP1 expression was also monitored at E18.5 in *Hoxa5* null embryos. UCP1 was shown to be expressed and active in uncoupling the proton gradient in brown adipocytes by E17.5 (Mayeuf-Louchart et al., 2019). UCP1 expression distribution appeared similar between *Hoxa5* mutant and control embryos (Figures 5E,J).

Finally, we also tested *Hoxa5* null BAT for transcriptional changes. sBAT was dissected from E18.5 embryos and qRT-PCR was performed for key markers of fat and muscle differentiation (Figure 5L). Most markers were expressed at levels statistically indistinguishable from controls. However, three markers associated with mitochondrial abundance and specialization in BAT, were significantly upregulated. These included *Ucp1* as well as *Ppargc1a*, encoding a key transcriptional regulator of mitochondrial biogenesis, and *Cox7a1*, encoding a component of the mitochondrial respiratory chain. Several additional adipocyte markers showed a non-significant trend toward increase, including both pan-adipocyte and brown-specific transcriptional regulators, and markers for fatty acid uptake and lipolysis. White adipocyte markers showed no mean difference across genotypes, while markers for pre-adipocytes and for skeletal muscle both showed a non-significant trend toward decrease. Together, this suggested that *Hoxa5* null sBAT, although morphologically smaller, is transcriptionally similar to wild-type sBAT.

Variation in the level of UCP1 protein was apparent among individual adipocytes, in both genotypes, with some cells appearing to express much higher levels than others by IF (Figures 5E,J). The global transcriptional increase of *Ucp1* and other mitochondrial markers in whole *Hoxa5* null BAT, particularly coupled with the decrease in adipocyte density, indicates that individual *Hoxa5* null adipoctyes express higher levels of these genes. Whether this could reflect some variation in previously unidentified subtype heterogeneity among adipocytes at embryonic stages, or is due to variation in the differentiation states of cells within a single adipocyte population is not clear. qRT-PCR on *Hoxa5* null sBAT revealed no effect on embryonic expression of recently-identified markers for subtypes of adult brown adipocyte (Karlina et al., 2021), including *Bin1, Tcf24, Eif5* or *P2rx5* (not shown).

### *Hoxa5* Null BAT and Epaxial Muscle Phenotypes Persist in Adults

We next wished to determine whether BAT phenotypes observed in E18.5 embryos persisted in adults. Although virtually all *Hoxa5^–/–^* animals die at birth from respiratory arrest (Jeannotte et al., 1993), rare individuals escape this lethal phenotype. We collected four escapers along with co-housed same-sex littermates, and aged them over a range from 2 months (young adult) to 18 months (aged adult). We dissected BAT depots as well as one representative epaxial muscle, the semispinalis capitus. The weight of each organ was measured, and each was photographed in whole-mount. In 4/4 pairs, iBAT and sBAT were reduced in *Hoxa5^–/–^* animals by relative weight, and this reduction was also visually apparent (Supplementary Table 3 and Figures 6A–C). cBAT was not significantly different from controls in weight or photographed area.

As previously reported (Mo et al., 2017), iBAT and sBAT grow during adulthood. In our small sample, we found the same trend (Figures 6A,B, compare the 2- and 6-month samples), and that these organs decreased in size in aged animals (18 months). Although this temporal pattern was observed in both genotypes, the BAT reduction in *Hoxa5* mutants compared to controls was more extreme in older animals, suggesting that it worsens with time. Conversely, the semispinalis capitus muscle was both heavier (Figure 6C) and thicker in both the dorsal-ventral and medio-lateral planes (Supplementary Figure 5), in 3/4 and 2/3 *Hoxa5* null adults compared to controls.

The cellular defects of *Hoxa5* null BAT also persisted in adults, including reduced adipocyte density and reduced and disorganized lipid droplets (Figures 6D–M). Together, this indicated that *Hoxa5* loss-of-function leads to persistent defects in iBAT and sBAT as well as to changes in epaxial skeletal muscle pattern from late embryos throughout adult life.

### The *Hoxa5* BAT and Epaxial Skeletal Muscle Phenotypes Can Be Recapitulated by *Hoxa5* Deletion in the *Myf5* Expression Domain

We previously showed that HOXA5 is not expressed in skeletal muscle or in the skeletal muscle lineage (Landry-Truchon et al., 2017; Holzman et al., 2018). This raised the question of whether these phenotypes arise from an autonomous requirement for *Hoxa5* in BAT or in BAT and skeletal muscle progenitors, or if instead they are a secondary consequence of the loss of HOXA5 activity in neighboring structures, such as cartilage or connective tissue. To test this, we conditionally deleted *Hoxa5* from *Myf5*-expressing cells using a *Myf5*^Cre^ mouse line (Tallquist et al., 2000). We confirmed the previously-reported activity of *Myf5/Cre* in both brown adipocytes and skeletal muscle in our crosses (Seale et al., 2008; Sanchez-Gurmaches and Guertin, 2014b; not shown). We used a conditional, *Hoxa5*^fl^ allele in which Cre recombinase activity was shown to generate an amorphic allele that abrogates *Hoxa5* expression and recapitulates *Hoxa5* null phenotypes (Tabariès et al., 2007). We also confirmed the spatial pattern of *Hoxa5* conditional deletion using immunofluorescence at E16.5 (Supplementary Figure 4), the latest stage at which HOXA5 protein is reliably detected (Figure 1). As expected based on previous reports of *Myf5/Cre* activity (Seale et al., 2008; Sanchez-Gurmaches and Guertin, 2014b), virtually all adipocytes in iBAT and sBAT lacked nuclear HOXA5 signal indicating efficient deletion. At this stage, we did not observe cBAT as a distinct structure, so we could not assess HOXA5 expression there. In contrast, many connective tissue fibroblasts of both BAT and muscle retained HOXA5 expression, consistent with our finding that many of these fibroblasts are *Myf5/Cre* negative (not shown). As a positive control, we detected typical HOXA5 expression in the nuclei of tracheal chondrocytes, a cell type in which *Myf5* is not expressed (Supplementary Figure 4N).

Next, BAT and skeletal muscle areas were compared in sections of *Myf5^Cre/^*^+^*; Hoxa5 ^fl/fl^* embryos vs. littermate *Myf5^Cre^; Hoxa5^fl/^*^+^ controls at E18.5. Conditional *Hoxa5* deletion completely recapitulated the reduced BAT area apparent in tissue sections, measured and combined for all three depots from C6-T1, as described for the null embryos above (Figures 7A,B; compare to Figures 4A,B). On average, BAT area was reduced to 73% of littermate controls (*p* = 0.0125, paired one-tailed *t*-test; *n* = 3 embryo pairs), similar to the reduction to 69% observed in *Hoxa5* null embryos (Figure 7C). Conditional *Hoxa5* deletion in the *Myf5* expression domain also led to the expanded epaxial muscle area observed in null mutants, although this expansion was less severe than that observed in null embryos (compare Figures 7D,E,H,I with Figures 4C,D,G,H). We did not observe a reduction in hypaxial muscles as we detected in null embryos (Figures 7F,G,J,K).

### *Hoxa5* Perturbs Early Stages of BAT Development, but Is Not Required to Repress Skeletal Muscle Fate

We next assessed whether the *Hoxa5* null BAT and muscle phenotypes could be observed earlier in embryogenesis. At E14.5, the first stage when BAT is morphologically distinct, we observed smaller BAT anlage and reduced density of PPARγ-positive adipocytes in *Hoxa5* null mutants (Figures 8A–D). This suggested that at least a part of *Hoxa5’s* role in BAT development occurs prior to E14.5.

We therefore examined expression of early adipocyte markers by qRT-PCR in C3-T2 trunk (the segments corresponding to the *Hoxa5* expression domain), which had been dissected from the neural tube, limbs, and thoracic organs to enrich for somite-derived tissue. As shown in Figure 8E, *Ebf2* mRNA, encoding the earliest known BAT marker, showed a trend toward decrease at E12.5 but not at E13.5. *Prdm16* mRNA showed no difference at E12.5 (the stage its expression was reported to initiate), but at E13.5 was significantly reduced in *Hox5* null segments compared to controls. However, as shown in Figure 2, both *Ebf2* and *Prdm16* show broad expression beyond prospective BAT. We therefore also assessed *Fabp4* which is highly but not exclusively expressed in differentiating adipocytes, and whose promoter can be activated by HOXA5 in adult pre-adipocytes (Cao et al., 2018b). *Fabp4* mRNA was downregulated at both stages, and significantly decreased at E13.5 (Figure 8E).

These results indicate that *Hoxa5* phenotypes are apparent early, and thus *Hoxa5* may act on BAT development as early as somite stages. Indeed somitic expression of HOXA5 is first detected between E9.5–E10.5 (Holzman et al., 2018), and the BAT and skeletal muscle lineages are known to have separated by E11.5 (Lepper and Fan, 2010). This led us to hypothesize that *Hoxa5* could promote BAT cell fate and repress skeletal muscle fate in their common progenitor within dermomyotome. Alternatively, *Hoxa5* could act downstream of BAT fate specification and affect the expansion, survival or differentiation of BAT progenitors. To test the first hypothesis, we used *Hoxa5* loss of function embryos carrying *Hoxa5/Cre* to ask whether the *Hoxa5* lineage ectopically gave rise to muscle in the absence of HOXA5 activity. This experiment was possible because the *Hoxa5* lineage does not normally contribute to skeletal muscle in wild-type embryos (Holzman et al., 2018). We generated embryos carrying the *Tg^Hoxa5Cre^; Rosa26^nYFP^*; *Hoxa5^–/–^* genotype and compared them to *Tg^Hoxa5Cre^; Rosa26^nYFP^ Hoxa5*^+/–^ control littermates. In both genotypes, cells with a history of activating the *Hoxa5* promoter express Cre, labeling them and their descendants with YFP. Interestingly, these *Hoxa5* lineage-labeled cells showed the same distribution in *Hoxa5* null embryos as in controls (Figures 8F–I). Namely, *Hoxa5* descendant cells were found in dermis, cartilage, BAT, and connective tissue including MCT fibroblasts within skeletal muscle, but not in skeletal muscle itself. This suggests that *Hoxa5* is not required to repress skeletal muscle specification in a common progenitor of muscle and BAT.

*Hoxa5* may act early to regulate the selective expansion or survival of BAT progenitors or preadipocytes. Labeling proliferating cells with PCNA revealed no obvious change in the abundance of proliferating cells in E11.5–E12.5 somites or at E14.5, the first stage where we can observe reduced cell density in *Hoxa5* null BAT (Supplementary Figure 6). Virtually all cells at these stages were PCNA positive. Co-labeling the *Hoxa5* lineage with *Hoxa5/Cre* as described above allowed us to focus on *Hoxa5* expressing cells in E11.5–E12.5, but again no difference was detected between genotypes. Labeling apoptotic cells with cleaved Caspase 3 similarly showed very few apoptotic cells and no differences between genotypes in embryos in E11.5–E12.5 somites (Supplementary Figures 6A–H), or at E14.5 or E18.5 (Supplementary Figures 6I–P), suggesting that *Hoxa5* is not required for adipocyte survival.

In summary, morphological and gene expression changes show an early role for *Hoxa5* in BAT development that may contribute to the phenotypes observed at birth. Interestingly, *Hoxa5* null embryos also present perturbed lipofibroblast marker expression in lungs (Landry-Truchon et al., 2017), leading us to investigate whether the effects we observed in somites might represent common molecular targets across different tissue types. qRT-PCR at E18.5 revealed that both *Prdm16* and *Fabp4* were significantly reduced in *Hoxa5* null lungs and diaphragm but not in trachea (Figure 8E). Together, this indicates that positive regulation of adipocyte development may be a common regulatory pathway for *Hoxa5* across different tissue types.

## Discussion

### *Hoxa5* Plays a Non-redundant Role in BAT Development

BAT is well-documented for its role in mammalian thermogenesis and metabolism, and for its influence on human conditions including obesity and diabetes. However, its developmental origins are incompletely described and only a handful of genes are known to cause embryonic BAT phenotypes once mutated. Molecular insight into the development of BAT can shed light on its post-natal mechanisms and relationship to human health and disease, and can inform therapeutic strategies.

Our results show that *Hoxa5* positively regulates the development of sBAT and iBAT, with molecular differences apparent as early as E13.5, cellular changes in differentiation characteristics, and with reduced BAT area in embryos. sBAT and iBAT reduction was concomitant with expanded epaxial muscles, and these reciprocal phenotypes persisted in adulthood. The embryonic BAT reduction was subtle and most clearly observed in tissue sections, where the morphological change was consistent and significant within the domain we measured (C6-T1). However, the reduction appeared less pronounced when BAT depots were removed and examined in whole-mount. We cannot readily account for this difference, but it is possible that the expanded epaxial musculature plays a role constraining the spatial domain BAT occupies *in situ.* Although the entire domain was examined, it is also possible that the most severe effects are localized to C6-T1, which is where the bulk of the sBAT is located, just beneath the scapular blade. However, we note that the same trend toward reduced sBAT and iBAT was observed in all measurements including whole mount and section.

Although overall BAT reduction was subtle, few genes are known to have a non-redundant effect on embryonic BAT, and further study of *Hox-*mediated patterning will be important to characterizing its embryonic origin. Further, the phenotypes described for each of the key players are overlapping but not identical. For example *Prdm16* is largely dispensable for BAT development, and either *Ebf2* mutant or *Prdm16; Prdm3* double mutant BAT develop largely normally but take on characteristics of WAT *in vivo* that becomes more extreme postnatally and ectopically transcribe myogenic transcripts in BAT (Seale et al., 2008; Rajakumari et al., 2013; Harms et al., 2014; Wang et al., 2014). In contrast, we did not observe WAT features in *Hoxa5* null BAT. Mouse mutants for either *Ews* or *Bmp7* show substantially reduced BAT area at birth (Tseng et al., 2008; Park et al., 2013), and the latter were most dramatically affected, with 50-70% reduction in iBAT compared to controls (Tseng et al., 2008). In comparison, *Hoxa5* null BAT also showed a consistent but less extreme reduction in iBAT and sBAT evident *in situ*. However, while *Bmp7* mutant embryos showed reduced lipogenesis and reduced *Ucp1* mRNA and protein expression, *Hoxa5* mutant BAT showed lipid droplet disruption but a slight increase in mitochondrial differentiation markers *Ucp1* and *Ppargc1a*. Further, unlike phenotypes for many of the genes above, *Hoxa5* null BAT did not show enhanced myogenic transcription.

Interestingly, a more extreme reciprocal reduction in BAT with expansion of epaxial skeletal muscle was reported in *TAF7*L^–/–^ neonates (Zhou et al., 2014). *TAF7L* encodes an alternative subunit of TFIID that interacts directly with PPARγ to regulate promoters of BAT-differentiation genes. Nothing further is known about the mechanism of its action in this context, however, the similarity in phenotypes raises the question of whether HOXA5 is also a member of this coactivator complex, and/or its transcriptional target.

The early molecular effects, BAT reduction and reduced adipocyte density observed in *Hoxa5* null embryos as early as E13.5–E14.5 led us to hypothesize that it is integrated into the genetic circuits that specify BAT in cervical and brachial somites. However, *Hoxa5* was not necessary to repress skeletal muscle fate in *Hoxa5* expressing cells, as shown by *Hoxa5/Cre* lineage mapping in *Hoxa5* null embryos. This indicates that it is dispensable for a BAT/skeletal muscle lineage decision within somites. It is possible that HOXA5 acts redundantly with additional HOX or other transcription factors to promote BAT specification at the expense of muscle in cervical dermomyotomes. Indeed, several *Hox4-5* transcripts are co-expressed with *Hoxa5* in cervical and brachial somites [reviewed in Mallo et al. (2010)]. Alternatively, *Hoxa5* may not be involved in the BAT/skeletal muscle lineage switch and instead may act solely downstream of adipocyte specification. For example, *Hoxa5* could affect the proliferation or survival of adipocytes or their progenitors. We did not detect obvious differences in cell proliferation or apoptotic cells from *Hoxa5* null somites or in BAT. However, these analyses by IF did not measure the cell division rate, and could not reveal a subtle change in the numbers of dividing cells that could be sufficient to explain the 30% reduction in BAT cross-sectional area that we observed.

Interestingly reduced *Fabpb4* expression was detected in multiple *Hoxa5* null tissues. When combined with a previous finding that in lung, additional lipofibroblast markers are also reduced in *Hoxa5* null embryos (Landry-Truchon et al., 2017), this suggests a conserved role for *Hoxa5* in positive regulation of adipocyte fate across multiple tissues.

### Roles for HOXA5 in Adipocyte Differentiation

Previous reports showed that *Hoxa5* overexpression in adult WAT primary cell culture promoted adipocyte differentiation as measured by marker expression, lipid droplet accumulation, and mitochondrial content (Cao et al., 2018b), as well as white adipocyte browning (Cao et al., 2018a). Conversely, RNAi-knockdown of *Hoxa5* in adult WAT cultures led to reduced expression of adipose markers and reduced lipid droplets. Therefore, these authors hypothesized that *Hoxa5* promotes the transition of preadipocytes to adipocytes (Cao et al., 2018b).

*Hoxa5* embryonic expression and null phenotypes are largely but not entirely consistent with such a role in embryonic BAT. HOXA5 protein expression initiates in somites between E9.5–E10.5 (Holzman et al., 2018). Approximately 1 day later, at E11.5, EBF2 expression is first reported in somites (Wang et al., 2014), and BAT has definitively separated from the muscle lineage, based on *Pax7/Cre* lineage mapping (Lepper and Fan, 2010). At E12.5, we found that HOXA5 is co-expressed with both EBF2 and nuclear PRDM16 specifically in a domain of cells antero-lateral to the epaxial muscle progenitors. We hypothesize that these cells include BAT progenitors. Since both EBF2 and PRDM16 are broadly expressed and not limited to BAT, we cannot definitively say that these co-expressing cells are prospective BAT. However, their position is consistent with lineage-mapping reports that BAT is derived from cells expressing markers for central-dorsal dermomyotome including *En1* and *Pax7*. More work, including identification of early BAT molecular markers, is needed to definitively locate BAT progenitors in somites.

HOXA5 expression is uniformly high in BAT from E14.5–E16.5, but becomes downregulated from E16.5–E18.5. This downregulation is coincident with the appearance of differentiated adipocyte features in embryonic BAT depots, including lipid droplet formation and UCP1 expression, both of which were reported to commence between E16.5–E17.5 (Mayeuf-Louchart et al., 2019). In E18.5 embryos and adults, we could only detect expression of *Hoxa5* using a *Hoxa5/Cre-GFP* transgene, and it was only present in a subset of adipocytes, all of which being also PPARγ positive.

*Hoxa5* null BAT from embryos, neonates and adults showed reduced lipid droplets. This also fits with reduced lipogenesis in primary adult adipocytes cultures following *Hoxa5* depletion (Cao et al., 2018b). However, that study also found a reduction in all BAT differentiation markers including *PPARgc1a* and *Ucp1* mRNA and protein. In contrast, we found slight but significantly increased expression of *PPARgc1a* and *Ucp1* mRNA, markers of mitochondrial biogenesis and BAT-specific differentiation.

Overall, our qRT-PCR panel of markers for various cell types indicated that the embryonic sBAT of *Hoxa5* mutants has a transcriptional profile similar to controls, although *Ucp1* and other mitochondrial markers were slightly upregulated. This suggests that once BAT depots have formed, their development is relatively normal. However, persistent changes in lipid droplet accumulation at all stages were observed. It will be important in the future to characterize the physiological impact of these differences in post-natal BAT, for example following a conditional knockout to bypass the perinatal lethal phenotype.

scRNA-seq profiling recently revealed that adult BAT contains a heterogeneous population of adipocytes: some with higher *Ucp1* and other thermogenic (mitochondrial and lipogenic) markers, and others characterized by lower *Ucp1* and increased markers for fatty acid uptake (Song et al., 2020). However, only high-*Ucp1* expressing subtypes were found until a few days after birth (Song et al., 2020), and we did not find significant differences in any subtype markers other than *Ucp1* in *Hoxa5* null embryos. It would be interesting to determine whether some of the recently discovered heterogeneity among BAT adipoctyes does develop pre-natally (Song et al., 2020; Karlina et al., 2021).

While more work is needed to identify the direct and indirect targets of *Hoxa5* in BAT, the phenotypes and expression of *Hoxa5* at many stages, suggest plays multiple roles in BAT development and maintenance.

### *Hoxa5* Acts Within the *Myf5* Lineage to Pattern BAT and Epaxial Skeletal Muscles

Conditional deletion of *Hoxa5* in the *Myf5* lineage recapitulated the BAT reduction and epaxial muscle expansion observed in null embryos. This shows that these phenotypes owe to an autonomous role for HOXA5 in the dermomyotome and its derivatives, rather than a secondary effect of HOXA5 activity in other lineages, such as skeleton. Notably, *Myf5/Cre* efficiently removed HOXA5 expression from adipoctyes, but many fibroblast connective tissue cells of both BAT and muscle retained HOXA5 expression. In combination with the previous finding that HOXA5 is not expressed in skeletal muscle, or in skeletal muscle progenitors (Landry-Truchon et al., 2017; Holzman et al., 2018), we therefore hypothesize that HOXA5 acts cell-autonomously in adipocytes or their progenitors, although a role in fibroblasts cannot be ruled out. Further, *Hoxa5* null mutants showed reduced sBAT and iBAT, but no reduction in cBAT and even a small but significant increase apparent by weight in embryos. It may be relevant in this context that most adipocytes in iBAT and sBAT are derived from the *Myf5* lineage, but over half of cBAT adipocytes are not [reviewed in Sanchez-Gurmaches and Guertin (2014a)], so they may be under different developmental control. In addition, cBAT is formed partially anterior to the *Hoxa5* expression domain. However, the precise somites that contribute to the BAT depots have yet to be mapped and the other embryonic tissue(s) that contribute adipoctyes to cBAT are unknown.

In addition to expanded epaxial muscle area, *Hoxa5* null mutants show a reduction of hypaxial muscles, raising the question of how *Hoxa5* could mediate these opposite effects. However, epaxial and hypaxial muscles are known to be regulated by separable pathways, and thus this opposite effect may reflect two or more different roles for *Hoxa5.* For example, epaxial and hypaxial muscle are derived from different regions of the dermomyotome (central/dorsal and ventral, respectively), and several mutations are known to preferentially decrease hypaxial muscles with no or milder effects on epaxial muscles {for example, *MyoD*, *Paraxis*, *Eya1*, *Six1*, *Pax3, Tbx1;* [reviewed in Buckingham and Rigby (2014); Heude et al. (2018)]}. Conversely, mutations in *Myf5* delay the development of epaxial muscles, but do not affect development of hypaxial muscles (Rudnicki et al., 1993). Thus, these two populations are under distinct developmental controls. However, in both cases, regulation of muscle development by *Hoxa5* is not cell-autonomous, because HOXA5 is not expressed in skeletal muscle, nor does the *Hoxa5* lineage contribute to skeletal muscle [(Landry-Truchon et al., 2017; Holzman et al., 2018) and this work].

In contrast to the effect on epaxial muscles, conditional deletion of *Hoxa5* in the *Myf5* domain did not reproduce the hypaxial muscle phenotypes observed in null embryos. It is possible that *Hoxa5* activity outside of the *Myf5* domain mediates its muscle patterning activity. The reduced BAT area itself could secondarily alter the area occupied by epaxial muscles. Alternatively, *Hoxa5* may act from within the *Myf5* lineage to pattern one or both populations (epaxial and hypaxial), but incomplete and/or late deletion with the *Myf5/Cre* line could be insufficient to produce muscle phenotypes. Of particular note, muscle connective tissue fibroblasts highly express HOXA5 (Landry-Truchon et al., 2017; Holzman et al., 2018) and are a potential source for patterning activity. Innervation defects have also been implicated as a cause for thinner myofibers of *Hoxa5* null diaphragm muscle (Landry-Truchon et al., 2017).

### A Role for *Hox* Genes in the Origin of BAT

Endothermy has evolved many times in vertebrates, and proceeds by varied mechanisms (Jastroch and Seebacher, 2020; Legendre and Davesne, 2020). Thermogenic adipoctyes, and the presence of brown adipose tissue depots, are unique to placental mammals. Physiologically and developmentally, brown adipocytes are thought to be most closely related to skeletal muscle, and the genetic circuitry that underlies their development from dermomyotome progenitors likely evolved via modification of a skeletal muscle differentiation program [reviewed in Oelkrug et al. (2015); Jastroch et al. (2018)]. The major BAT depots, iBAT, sBAT and cBAT, form around the cervical and brachial segments, and thus at a specific axial level. Clearly, the thermogenic adipoctye differentiation program can be deployed in multiple regions of the body, and from different cell types with varied developmental histories. However, the axial position and somite origin of the major depots makes *Hox* group 4–5 genes candidates both for their positioning and development from dermomyotome progenitors; such an evolutionarily derived role would be an interesting area for future study. *Hoxa5* was previously described as part of the brown adipocyte gene expression signature (Wang et al., 2014), and we found it is present in dermomyotome from stages prior to the specification of BAT. *Hoxc4* and *Hoxc8* are also expressed in differentiating adult brown adipocytes (Singh et al., 2016). To our knowledge, embryonic *Hox* expression or function in BAT, except for HOXA5 (Holzman et al., 2018) has not been characterized. Future work studying these and other aspects of the evolutionary origin of the BAT circuit can shed light on the evolutionary and developmental origins of this important tissue.

## Data Availability Statement

The original contributions generated for this study are included in the article/Supplementary Material, further inquiries can be directed to the corresponding author/s.

## Ethics Statement

The animal study was reviewed and approved by Columbia University IACUC.

## Author Contributions

JM, MH, and LJ designed the study. MH, AR, TF, KL-T, KS, JB, and JM conducted the experiments. JM wrote the manuscript. All authors contributed to the article and approved the submitted version.

## Conflict of Interest

The authors declare that the research was conducted in the absence of any commercial or financial relationships that could be construed as a potential conflict of interest.

## Supplementary Material

Supplementary Figure 1 The axial position of major BAT depots at E18.5. Panels **(A–F)** show dorsal views of progressive stages of dissection of one embryo from dorsal to ventral. **(A)** Dorsal view following removal of the dermis reveals iBAT lobes, external to muscle layers. Yellow dotted line outlines the right iBAT lobe. Inset shows this right lobe flipped 90°C clockwise, in medial view (anterior is up, ventral to the left), which shows that the thickest part of the iBAT is in the anterior, with an apex at approximately T1–T2. C7 and T5 mark the anterior and posterior extend of iBAT. Note the iBAT is covered by a thin layer of WAT, but in medial view (inset) it can be seen that the WAT is superficial. **(B)** Following removal of the iBAT, the deeper BAT lobes are still covered by epaxial muscles and scapula. The right scapular blade is indicated by the bracket. Green asterisk marks the position at C7 where the scapular blades meet. Black arrowheads are in the same position as in **(A)**. **(C)** Removal of the trapezius and rhomboid muscles, and release of the connective tissue at the midline reveals the cBAT (right lobe outlined with red dotted line), and the edge of the sBAT (right lobe outlined with blue dotted line), the latter located medial to the scapular blade. C1 (gray arrowhead) is now visible as a white line beneath the cBAT. **(D)** Lateral opening of the scapular blades better shows the position of the cBAT (C1–C7) and sBAT (approximately C7-T3). **(E)** Removal of the cBAT allows unambiguous assignment of vertebral identities and thus axial position of BAT lobes. While only C1 and T2 are marked, gray arrowheads indicate the 7 cervical vertebrae, and white arrowheads the first two thoracic vertebrae. **(F)** Dissection of the sBAT away from the scapular blade reveals the shape of its medial edge (now pointing dorsally after dissection), which is the thickest part of it. **(G)** Schematic of BAT lobe positions based on panels **(A–F)**. The left side shows a dorsal view, with anterior up, and the forelimb to the right at the level spanning segments C7-T2. Segment positions are indicated. The right side shows a cross section, diagramming positions of each BAT depot along the DV axis. cBAT, cervical BAT; fl, forelimb; iBAT, interscapular BAT; nt, neural tube; sBAT, scapular BAT; sc, scapula; vb, vertebral body; C1–C7, cervical vertebrae 1–7; T1–T2, thoracic vertebrae 1–2.

Supplementary Figure 2 **(A)** Dry weight of dissected BAT depots at E18.5, relative to the total embryonic body weight. Lines show median values; sBAT weight is significantly reduced and cBAT is significantly increased in *Hoxa5* null embryos (^∗^*p* < 0.05, ^∗∗^*p* < 0.005 one-tailed *t*-test). **(B–D)** Hematoxylin and eosin staining of E18.5 sBAT shows nuclear density in a wild-type **(A)** compared to a *Hoxa5* null **(B)** littermate. **(C)** Nuclei were counted in 5 different fields of view per embryo, including those shown in **(A,B)**. Nuclear density was significantly reduced in the *Hoxa5* null littermate (^∗∗∗^*p* = 0.0007, two-tailed *t*-test). Scalebar: 25 μm.

Supplementary Figure 3 **(A–H)** iBAT phenotype in neonates at postnatal day 2 (P2) following conditional deletion of *Hoxa5* with *Meox1*^Cre^. This conditional deletion removes *Hoxa5* from somite derivatives but allows mutants to bypass lethal respiratory phenotypes and survive birth. Panels **(A,E)** show lower magnification. **(B–D)** and **(F–H)** compare higher power views of iBAT stained with PPARγ to reveal adipocytes and BODIPY to reveal lipid droplets. Note that while lipid droplets are larger and more abundant at this stage than at others observed, they are still smaller and less organized following *Hoxa5* conditional deletion relative to controls. Scale bars: **(A,E)**, 50 μm; all other panels, 20 μm.

Supplementary Figure 4 Conditional deletion of *Hoxa5* with *Myf5/Cre* leads to efficient ablation of HOXA5 expression in adipocytes but not in connective tissue fibroblasts of BAT or skeletal muscle **(A–G)** HOXA5 IF in control sBAT at E16.5 shows expression in all adipocytes (yellow arrows indicate examples of HOXA5-expressing nuclei), and in PDGFRα-positive connective tissue fibroblasts (yellow arrowheads indicate HOXA5-positive fibroblasts). Insets indicated in **(A)** are shown at higher magnification for sBAT **(C,D)** and for epaxial muscle **(E,F)**. **(H–N)** HOXA5 IF in a *Myf5/Cre* conditionally-deleted littermate shows the expected pattern of HOXA5 ablation. Neither the wide-view nor inset **(J,K)** contain adipoctyes with nuclear HOXA5 signal (white arrows show examples of HOXA5-negative adipoctyes), indicating efficient ablation. However, some connective tissue fibroblasts retain HOXA5 expression (yellow arrowheads) while others are negative (white arrowhead). This is consistent with a *Myf5Cre RFP* reporter assay showing that Myf5 Cre is not active in many connective tissue fibroblasts in this region (data not shown). Note the punctate cytoplasmic staining in adipocyte cytoplasm in all genotypes is autofluorescence, as confirmed in a null *Hoxa5^–/–^* sample **(O–S)**. As a positive control, we detect abundant nuclear HOXA5 expression in tracheal chondrocytes (yellow arrowheads with red outlines), an area where *Myf5/Cre* is inactive. This staining is absent from a *Hoxa5* null trachea (**S**, white arrowhead with red outline). **(T,AB)** Co-staining with HOXA5 and nuclear adiopctye marker EBF2 marker further confirms that HOXA5 expression is efficiently ablated in adipocotyes (white arrows show examples of EBF2-postive nuclei). Yellow arrow in **(T)**, and shown in inset **(V–X)** shows a single HOXA5-positive adipocyte in this field of view. Elongated cells are either connective tissue fibroblasts or endothelial cells, and many of these retain HOXA5 expression (yellow arrowheads) in BAT **(V,X)** and epaxial muscle **(Y,AA)**. Scale bar in **(A)** is 50 μM (and applies to **A,B,G,H,I,N,S,T,U**). Scale bar in **(C)** is 20 μM and applies to all other panels.

Supplementary Figure 5 Enlarged epaxial muscle *splenius capitus* persists in some *Hoxa5* null adults. Rare *Hoxa5*^–^*^/^*^–^ animals that survived embryonic lethality were compared to *Hoxa5*^+^*^/^*^+^or ^±^ littermate controls. **(A–C)** Dissected *splenius capitus* muscles from control animals at the ages indicated are shown in dorsal view (left panels) medial view (right panels), anterior is up. **(D)** The same muscles are shown *in situ* for the 18 months stage, also in dorsal view. Arrowheads indicate the origin and insertion. Panels **(E–H)** show the same for *Hoxa5* null animals. In each image pair, white (control) and yellow (*Hoxa5* null) lines compare the muscle width at its maximum. Note that the 2 and 18 months specimens show enlarged muscles in nulls, while the 6 months specimen does not. Scale bars: 5 mm (**A–C,E–G** are the same scale, and **D,H** are the same scale).

Supplementary Figure 6 Abundance of proliferating cells in control **(A–G)** compared to *Hoxa5* null **(H–N)** embryos. Between E11.5–E14.5, virtually all somitic cells and derivatives are marked with PCNA indicating they are proliferative. Boxed areas in **(A,H)** are magnified in **(B,I)** and boxed areas in **(C,J)** are magnified in **(D,K)**. Embryos in E12.5 were of the full genotypes: *Tg*^Hoxa5Cre^*; Hoxa5*^+/+^*; Rosa26^nYFP/^*^+^; and *Tg*^Hoxa5Cre^*; Hoxa5*^–/–^*; Rosa26*^RFP/+^ such that cells activating the *Hoxa5* promoter could be visualized. Embryos at 12.5 were similarly labeled, except with the Rosa26RFP allele instead. There is also no obvious difference in the contribution of cells activating the *HoxA5* promoter (*Hoxa5* descendant cells) in wild type vs. *Hoxa5* null somites. Full genotypes for embryos shown in **(A–D,H–K)** activating the *Hoxa5* promoter are labeled with nuclear YFP (nYFP) **(B,I)** or RFP **(C,D,J,K)** in the indicated colors. d, dorsal root ganglion; na, neural arch; nt, neural tube; sc, scapula). Scale bars: **(A,H)** 100 μm; **(B,I)** 50 μm; **(C,J)** 200 μm; **(D,K)** 50 μm; **(E,F,L,M)** 200 μm; **(G,N)** 50 μm.

Supplementary Figure 7 Cleaved caspase 3 IF reveals no difference in abundance or distribution of apoptotic cells in *Hoxa5* null embryonic somites or BAT compared to controls. Panels **(A–H)** compare littermates of the genotypes in which the *Hoxa5* lineage is marked, as described in Supplementary Figure 4. Panels **(I–P)** compare littermates at stages after the morphological emergence of BAT. Arrows indicate examples of rare, CCASP3-positive cells. Scale bars: 100 μm (panels **A–H**), 200 μm (panels **I–P**).

Supplementary Table 1 Primary antibodies used in this study. All could be used with standard permeabilization except ^∗^required antigen retrieval and ^∗∗^ required methanol permeabilization (see section “Materials and Methods”).

Supplementary Table 2 qRT-PCR primers used in this study (5′–3′).

Supplementary Table 3 BAT and epaxial muscle weights in *Hoxa5* null animals. Four *Hoxa5* null adults that escaped perinatal lethality were co-housed with a same-sex littermate to the age indicated. Organs were dissected without PBS and weighed. Relative body weight is the weight of the null animal/WT paired control. Relative organ weights were normalized to the difference in body weight as follows: normalized, relative organ weight = [null organ weight (g)/control organ weight (g)]/[null body weight (g)/control body weight (g)]. These normalized, relative values are also plotted in Figure 6C. cBAT, cervical BAT; capitus, semipinalis capitus; iBAT, interscapular BAT; mo, months; norm, normalized; rel, relative; sBAT, scapular BAT; wt, weight.
