# Supplementary figures and images for: HOXA5 Participates in Brown Adipose Tissue and Epaxial Skeletal Muscle Patterning and in Brown Adipocyte Differentiation
Source: Front Cell Dev Biol. 2021 Feb 25;9:632303. doi: 10.3389/fcell.2021.632303 (PMC7959767; doi:10.3389/fcell.2021.632303)

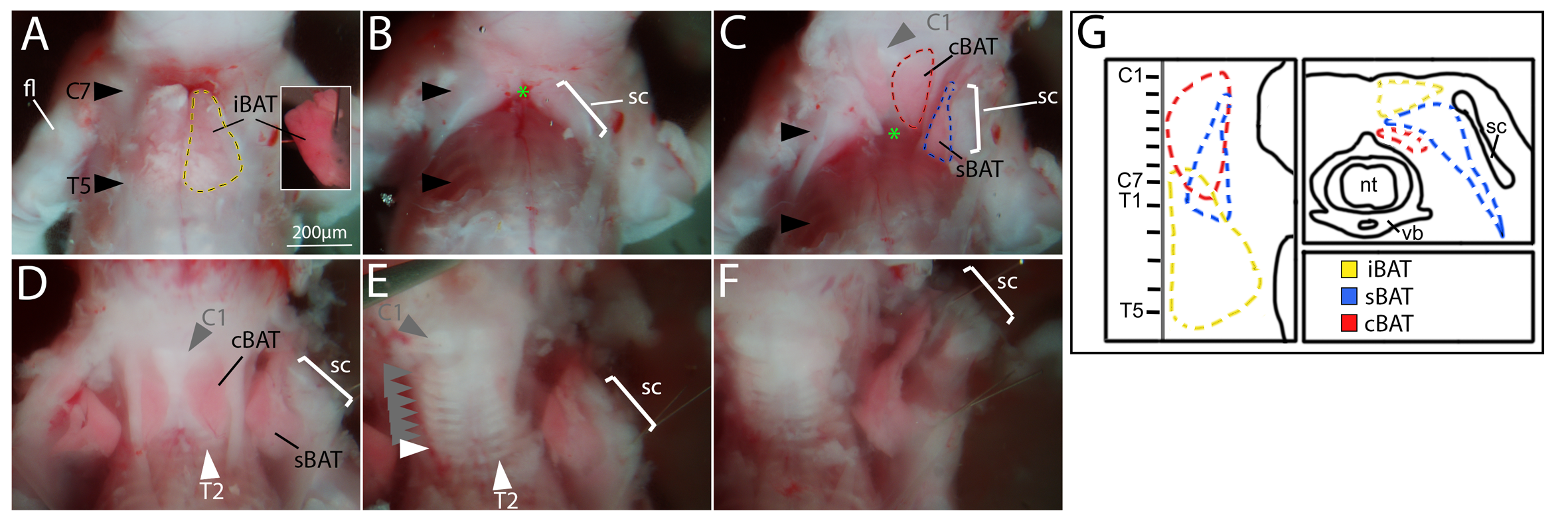

Supplement: Supplementary Figure 1 — The axial position of major BAT depots at E18.5. Panels (A–F) show dorsal views of progressive stages of dissection of one embryo from dorsal to ventral. (A) Dorsal view following removal of the dermis reveals iBAT lobes, external to muscle layers. Yellow dotted line outlines the right iBAT lobe. Inset shows this right lobe flipped 90°C clockwise, in medial view (anterior is up, ventral to the left), which shows that the thickest part of the iBAT is in the anterior, with an apex at approximately T1–T2. C7 and T5 mark the anterior and posterior extend of iBAT. Note the iBAT is covered by a thin layer of WAT, but in medial view (inset) it can be seen that the WAT is superficial. (B) Following removal of the iBAT, the deeper BAT lobes are still covered by epaxial muscles and scapula. The right scapular blade is indicated by the bracket. Green asterisk marks the position at C7 where the scapular blades meet. Black arrowheads are in the same position as in (A). (C) Removal of the trapezius and rhomboid muscles, and release of the connective tissue at the midline reveals the cBAT (right lobe outlined with red dotted line), and the edge of the sBAT (right lobe outlined with blue dotted line), the latter located medial to the scapular blade. C1 (gray arrowhead) is now visible as a white line beneath the cBAT. (D) Lateral opening of the scapular blades better shows the position of the cBAT (C1–C7) and sBAT (approximately C7-T3). (E) Removal of the cBAT allows unambiguous assignment of vertebral identities and thus axial position of BAT lobes. While only C1 and T2 are marked, gray arrowheads indicate the 7 cervical vertebrae, and white arrowheads the first two thoracic vertebrae. (F) Dissection of the sBAT away from the scapular blade reveals the shape of its medial edge (now pointing dorsally after dissection), which is the thickest part of it. (G) Schematic of BAT lobe positions based on panels (A–F). The left side shows a dorsal view, with anterior up, and the forelimb to [file Image_1.TIF]

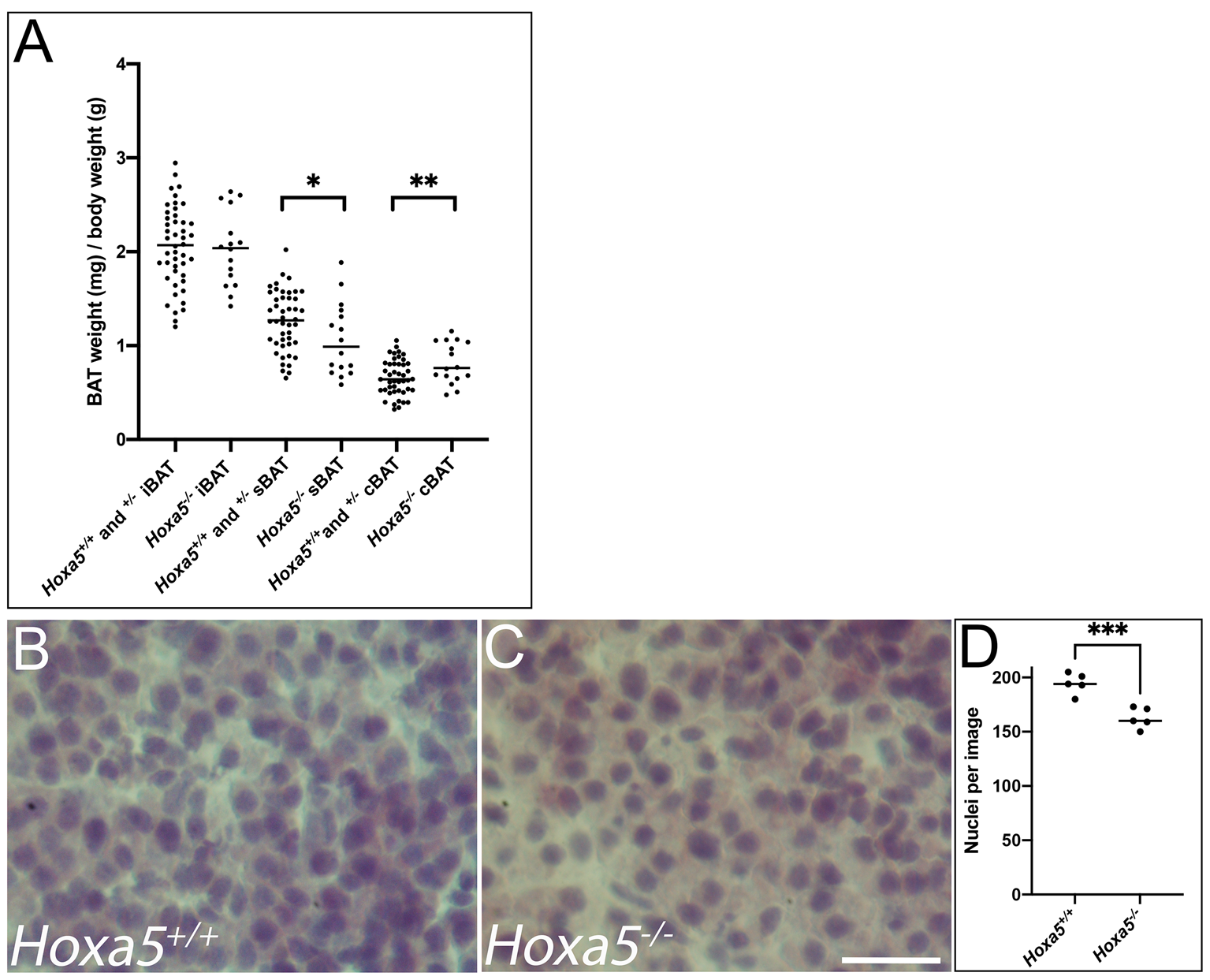

Supplement: Supplementary Figure 2 — (A) Dry weight of dissected BAT depots at E18.5, relative to the total embryonic body weight. Lines show median values; sBAT weight is significantly reduced and cBAT is significantly increased in Hoxa5 null embryos (∗p < 0.05, ∗∗p < 0.005 one-tailed t-test). (B–D) Hematoxylin and eosin staining of E18.5 sBAT shows nuclear density in a wild-type (A) compared to a Hoxa5 null (B) littermate. (C) Nuclei were counted in 5 different fields of view per embryo, including those shown in (A,B). Nuclear density was significantly reduced in the Hoxa5 null littermate (∗∗∗p = 0.0007, two-tailed t-test). Scalebar: 25 μm. [file Image_2.TIF]

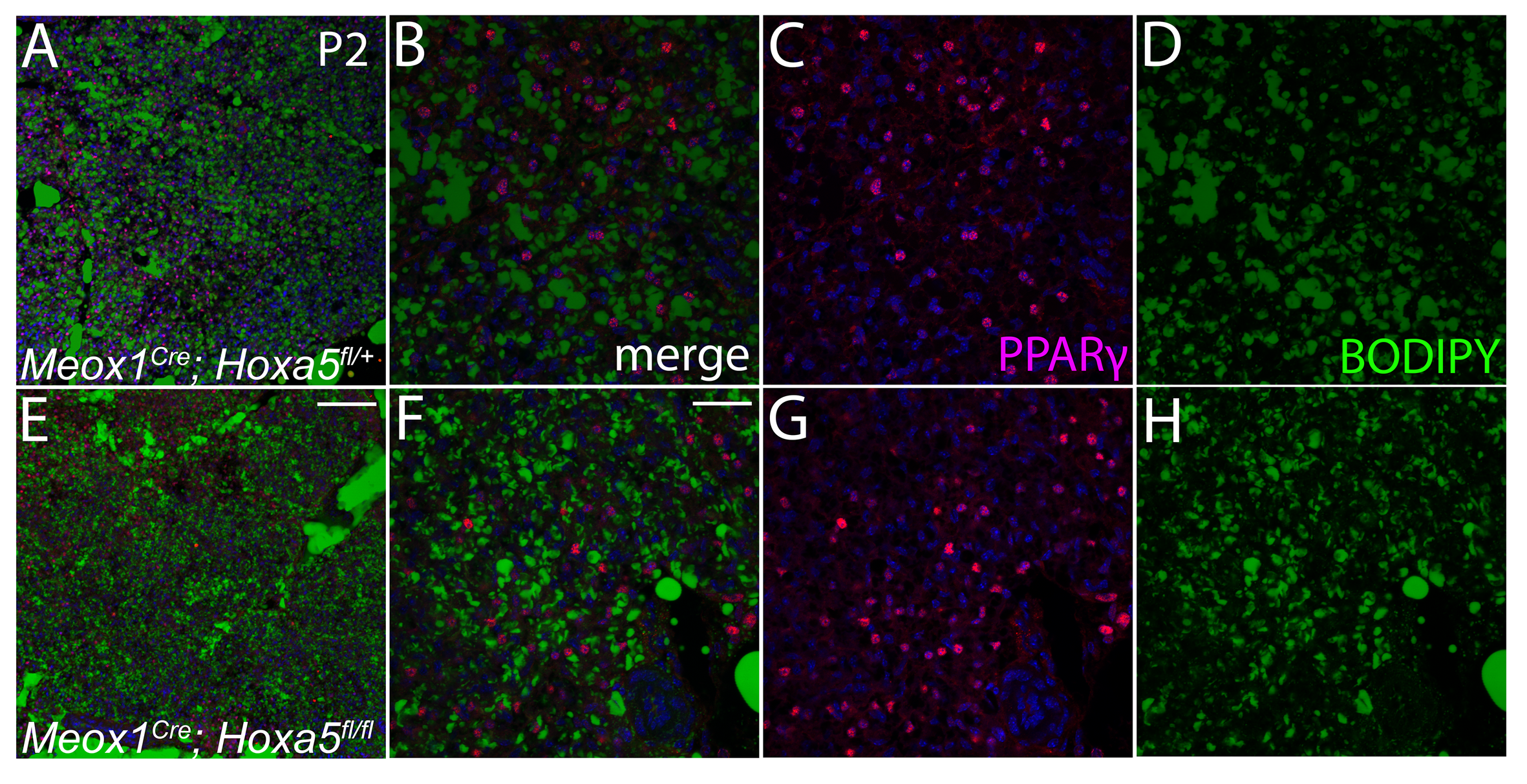

Supplement: Supplementary Figure 3 — (A–H) iBAT phenotype in neonates at postnatal day 2 (P2) following conditional deletion of Hoxa5 with Meox1Cre. This conditional deletion removes Hoxa5 from somite derivatives but allows mutants to bypass lethal respiratory phenotypes and survive birth. Panels (A,E) show lower magnification. (B–D) and (F–H) compare higher power views of iBAT stained with PPARγ to reveal adipocytes and BODIPY to reveal lipid droplets. Note that while lipid droplets are larger and more abundant at this stage than at others observed, they are still smaller and less organized following Hoxa5 conditional deletion relative to controls. Scale bars: (A,E), 50 μm; all other panels, 20 μm. [file Image_3.TIF]

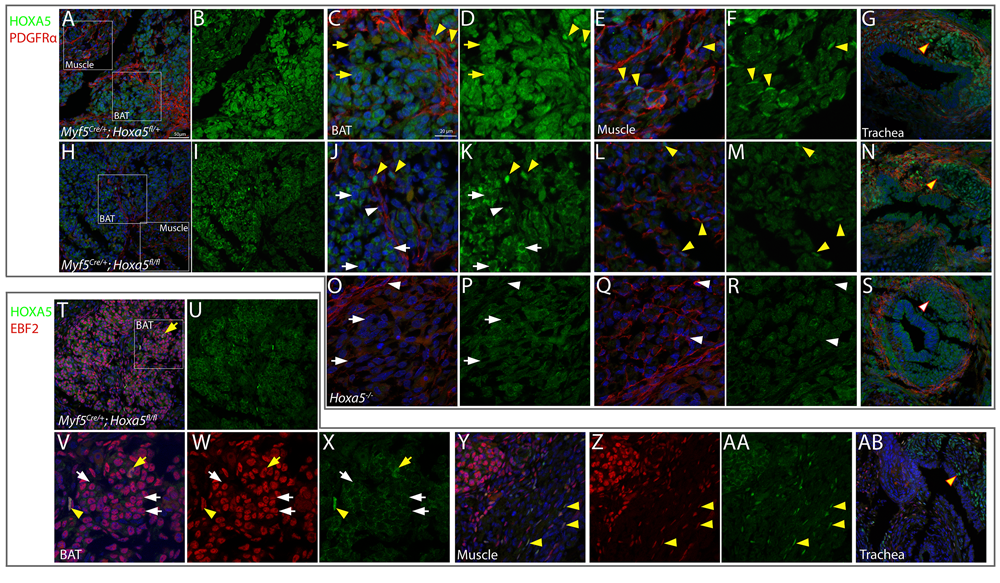

Supplement: Supplementary Figure 4 — Conditional deletion of Hoxa5 with Myf5/Cre leads to efficient ablation of HOXA5 expression in adipocytes but not in connective tissue fibroblasts of BAT or skeletal muscle (A–G) HOXA5 IF in control sBAT at E16.5 shows expression in all adipocytes (yellow arrows indicate examples of HOXA5-expressing nuclei), and in PDGFRα-positive connective tissue fibroblasts (yellow arrowheads indicate HOXA5-positive fibroblasts). Insets indicated in (A) are shown at higher magnification for sBAT (C,D) and for epaxial muscle (E,F). (H–N) HOXA5 IF in a Myf5/Cre conditionally-deleted littermate shows the expected pattern of HOXA5 ablation. Neither the wide-view nor inset (J,K) contain adipoctyes with nuclear HOXA5 signal (white arrows show examples of HOXA5-negative adipoctyes), indicating efficient ablation. However, some connective tissue fibroblasts retain HOXA5 expression (yellow arrowheads) while others are negative (white arrowhead). This is consistent with a Myf5Cre RFP reporter assay showing that Myf5 Cre is not active in many connective tissue fibroblasts in this region (data not shown). Note the punctate cytoplasmic staining in adipocyte cytoplasm in all genotypes is autofluorescence, as confirmed in a null Hoxa5–/– sample (O–S). As a positive control, we detect abundant nuclear HOXA5 expression in tracheal chondrocytes (yellow arrowheads with red outlines), an area where Myf5/Cre is inactive. This staining is absent from a Hoxa5 null trachea (S, white arrowhead with red outline). (T,AB) Co-staining with HOXA5 and nuclear adiopctye marker EBF2 marker further confirms that HOXA5 expression is efficiently ablated in adipocotyes (white arrows show examples of EBF2-postive nuclei). Yellow arrow in (T), and shown in inset (V–X) shows a single HOXA5-positive adipocyte in this field of view. Elongated cells are either connective tissue fibroblasts or endothelial cells, and many of these retain HOXA5 expression (yellow arrowheads) in BAT (V,X) and epaxial muscle (Y,AA). Scale bar [file Image_4.TIF]

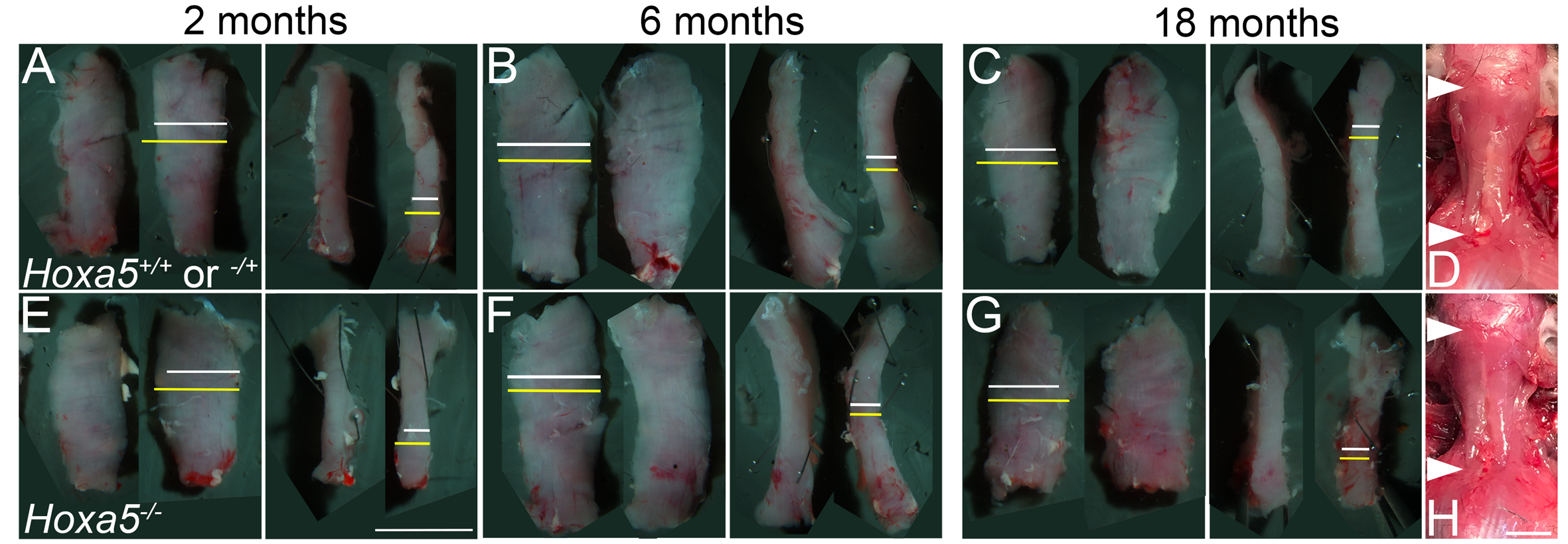

Supplement: Supplementary Figure 5 — Enlarged epaxial muscle splenius capitus persists in some Hoxa5 null adults. Rare Hoxa5–/– animals that survived embryonic lethality were compared to Hoxa5+/+or ± littermate controls. (A–C) Dissected splenius capitus muscles from control animals at the ages indicated are shown in dorsal view (left panels) medial view (right panels), anterior is up. (D) The same muscles are shown in situ for the 18 months stage, also in dorsal view. Arrowheads indicate the origin and insertion. Panels (E–H) show the same for Hoxa5 null animals. In each image pair, white (control) and yellow (Hoxa5 null) lines compare the muscle width at its maximum. Note that the 2 and 18 months specimens show enlarged muscles in nulls, while the 6 months specimen does not. Scale bars: 5 mm (A–C,E–G are the same scale, and D,H are the same scale). [file Image_5.TIF]

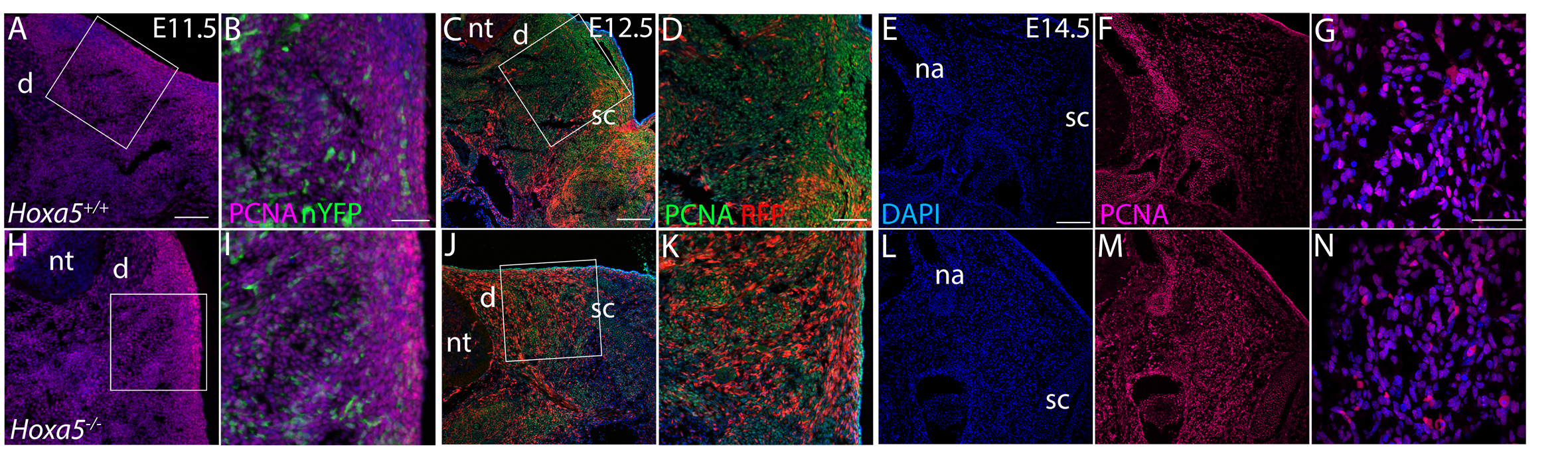

Supplement: Supplementary Figure 6 — Abundance of proliferating cells in control (A–G) compared to Hoxa5 null (H–N) embryos. Between E11.5–E14.5, virtually all somitic cells and derivatives are marked with PCNA indicating they are proliferative. Boxed areas in (A,H) are magnified in (B,I) and boxed areas in (C,J) are magnified in (D,K). Embryos in E12.5 were of the full genotypes: TgHoxa5Cre; Hoxa5+/+; Rosa26nYFP/+; and TgHoxa5Cre; Hoxa5–/–; Rosa26RFP/+ such that cells activating the Hoxa5 promoter could be visualized. Embryos at 12.5 were similarly labeled, except with the Rosa26RFP allele instead. There is also no obvious difference in the contribution of cells activating the HoxA5 promoter (Hoxa5 descendant cells) in wild type vs. Hoxa5 null somites. Full genotypes for embryos shown in (A–D,H–K) activating the Hoxa5 promoter are labeled with nuclear YFP (nYFP) (B,I) or RFP (C,D,J,K) in the indicated colors. d, dorsal root ganglion; na, neural arch; nt, neural tube; sc, scapula). Scale bars: (A,H) 100 μm; (B,I) 50 μm; (C,J) 200 μm; (D,K) 50 μm; (E,F,L,M) 200 μm; (G,N) 50 μm. [file Image_6.TIF]

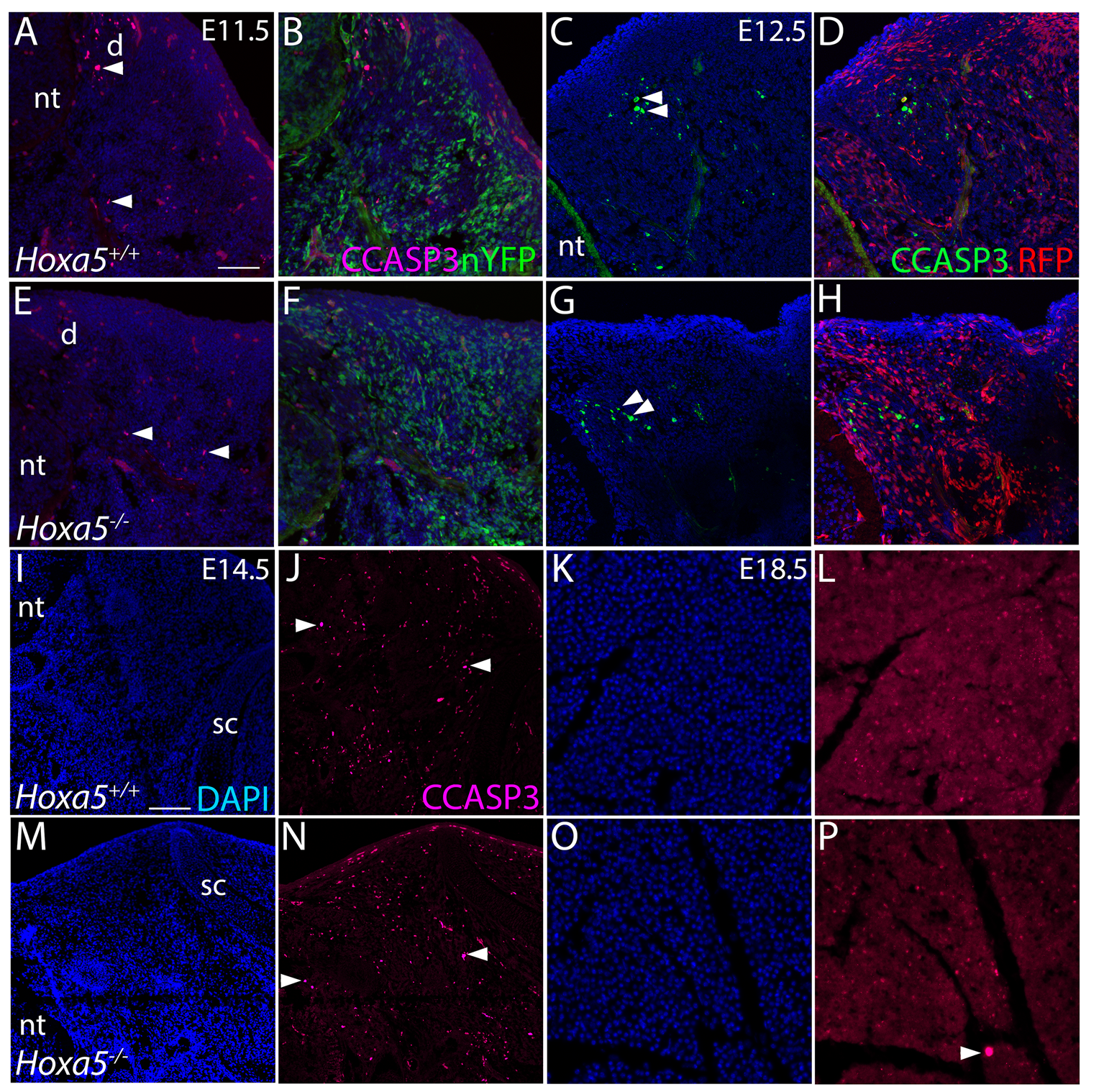

Supplement: Supplementary Figure 7 — Cleaved caspase 3 IF reveals no difference in abundance or distribution of apoptotic cells in Hoxa5 null embryonic somites or BAT compared to controls. Panels (A–H) compare littermates of the genotypes in which the Hoxa5 lineage is marked, as described in Supplementary Figure 4. Panels (I–P) compare littermates at stages after the morphological emergence of BAT. Arrows indicate examples of rare, CCASP3-positive cells. Scale bars: 100 μm (panels A–H), 200 μm (panels I–P). [file Image_7.TIF]
