# Supplementary material for: HOXA5 Participates in Brown Adipose Tissue and Epaxial Skeletal Muscle Patterning and in Brown Adipocyte Differentiation
Source: Front Cell Dev Biol. 2021 Feb 25;9:632303. doi: 10.3389/fcell.2021.632303 (PMC7959767; doi:10.3389/fcell.2021.632303)
Supplement: Supplementary Table 1 — Primary antibodies used in this study. All could be used with standard permeabilization except ∗required antigen retrieval and ∗∗ required methanol permeabilization (see section “Materials and Methods”). [file Table_1.docx]

| **Antigen** | **Antibody source** | **Dilution** | **species** |
| --- | --- | --- | --- |
| CCASP3 | CST 9661 | 1:100 | rabbit |
| EBF2 | RnD AF7006 | 1:13 | sheep |
| GFP/YFP | Abcam ab13970 | 1:500 | chicken |
| HOXA5 | (Dasen et al., 2005) | 1:500 | rabbit |
| Muscle Actin* | Abcam ab156302 | 1:100 | rabbit |
| Muscle Myosin4* | DSHB BF-F3 | 1:1 | mouse |
| PCNA | Santa Cruz sc-56 | 1:200 | mouse |
| PCNA** | CST 13110S | 1:400 | rabbit |
| PDGFRα | RnD af1062 | 15 µg/mL | goat |
| PERILIPIN | CST 9349 | 1:100 | rabbit |
| PPARγ | Thermo-Fisher MA5-14889 | 1:200 | rabbit |
| PRDM16 | RnD AF6295 | 1:13 | sheep |
| RFP | Rockland 600-401-379 | 1:200 | rabbit |
| UCP1 | Abcam ab10983 | 1:500 | rabbit |
