# Supplementary material for: HOXA5 Participates in Brown Adipose Tissue and Epaxial Skeletal Muscle Patterning and in Brown Adipocyte Differentiation
Source: Front Cell Dev Biol. 2021 Feb 25;9:632303. doi: 10.3389/fcell.2021.632303 (PMC7959767; doi:10.3389/fcell.2021.632303)
Supplement: Supplementary Table 2 — qRT-PCR primers used in this study (5′–3′). [file Table_2.docx]

| **Target gene** | **Forward primer** | **Reverse primer** |
| --- | --- | --- |
| *AdipoQ* | gcactggcaagttctactgcaaca | agagaacggccttgtccttcttga |
| *Atgl* | gtccttcaccatccgcttgtt | ctcttggccctcatcaccag |
| *Cd36* | agatgacgtggcaaagaacag | ggacctgatgcaaccctatga |
| *Cidea* | tgctcttctgtatcgcccagt | gccgtgttaaggaatctgctg |
| *Cox7a1* | cagcgtcatggtcagtctgt | agaaaaccgtgtggcagaga |
| *Cox8b* | gaaccatgaagccaacgact | gcgaagttcacagtggttcc |
| *Dio2* | cagtgtggtgcacgtctccaatc | tgaaccaaagttgaccaccag |
| *Ebf2* | ccaaacagttctgcaaaggag | attgtgtggtgtgccgtaga |
| *Elovl6* | tccgcgttctcatgtaggtct | ggacctgatgcaaccctatga |
| *Fabp4* | gcagacgacaggaaggtgaa | ccagcttgtcaccatctcgt |
| *Hoxa5* | cccagatctacccctggatg | ggcatgagctatttcgatcct |
| *Hsl* | tctatgcgcaggagtgtgtc | ttgacatcagagggtgtgga |
| *Igf1* | gaccagagaccctttgcgggg | tgaggtgccctccgaatgctg |
| *Myh3* | acgacaactcgtctcgcttt | ttggtcgtaatcagcagcag |
| *Myh8* | tgaccttgagctgacactgg | cttctccttggtcagcttgg |
| *Myogenin* | tccagtacattgagcgccta | acgatggacgtaagggagtg |
| *Nnmt* | ggagcctttgactggtcccca | cctgcttgattgcacgcctca |
| *Pdgfra* | ccatgcagttgccttacgact | agagcctgcttttcactagacc |
| *Pparg* | gccgagtctgtggggataaa | aggcacttctgaaaccgaca |
| *Ppargc1a* | ggagccgtgaccactgaca | tggtttgctgcatggttctg |
| *Prdm16* | agatgaaccaggcatccact | tcctgtgacttcccggcta |
| *Retn* | ctgtccagtctatccttgcacac | cagaaggcacagcagtcttga |
| *Rpl19* | gatcatccgcaagcctgtga | gcatccgagcattggcagta |
| *Ucp1* | tctcagccggcttaatgactg | ggcttgcattctgaccttcac |
