# Supplementary material for: HOXA5 Participates in Brown Adipose Tissue and Epaxial Skeletal Muscle Patterning and in Brown Adipocyte Differentiation
Source: Front Cell Dev Biol. 2021 Feb 25;9:632303. doi: 10.3389/fcell.2021.632303 (PMC7959767; doi:10.3389/fcell.2021.632303)
Supplement: Supplementary Table 3 — BAT and epaxial muscle weights in Hoxa5 null animals. Four Hoxa5 null adults that escaped perinatal lethality were co-housed with a same-sex littermate to the age indicated. Organs were dissected without PBS and weighed. Relative body weight is the weight of the null animal/WT paired control. Relative organ weights were normalized to the difference in body weight as follows: normalized, relative organ weight = [null organ weight (g)/control organ weight (g)]/[null body weight (g)/control body weight (g)]. These normalized, relative values are also plotted in Figure 6C. cBAT, cervical BAT; capitus, semipinalis capitus; iBAT, interscapular BAT; mo, months; norm, normalized; rel, relative; sBAT, scapular BAT; wt, weight. [file Table_3.docx]

| Genotype | Age  (months), Sex | | Body wt (g) | Relative body wt | iBAT wt (g) | Norm, Rel iBAT wt | sBAT wt (g) | Norm, Rel sBAT wt | cBAT wt (g) | Norm, Rel cBAT wt | capitus wt (g) | Norm, Rel capitus wt |
| --- | --- | --- | --- | --- | --- | --- | --- | --- | --- | --- | --- | --- |
| *Hoxa5^+/-^* | 2 , F | | 19.013 |  | 0.1042 |  | 0.0343 |  | 0.0259 |  | 0.043 |  |
| *Hoxa5^-/-^* |  | | 18.719 | 0.98 | 0.0987 | 0.96 | 0.0282 | 0.84 | 0.0159 | 0.62 | 0.0456 | 1.08 |
|  |  | |  |  |  |  |  |  |  |  |  |  |
| *Hoxa5^+/-^* | 6 , M | | 28.705 |  | 0.129 |  | 0.0508 |  | 0.0186 |  | 0.0672 |  |
| *Hoxa5^-/-^* |  | | 28.982 | 1.01 | 0.1092 | 0.84 | 0.0363 | 0.71 | 0.0248 | 1.32 | 0.075 | 1.11 |
|  |  | |  |  |  |  |  |  |  |  |  |  |
| *Hoxa5^+/+^* | 9 , F | | 28.705 |  | 0.2398 |  | 0.0767 |  | 0.0293 |  | 0.0779 |  |
| *Hoxa5^-/-^* |  | | 32.785 | 1.14 | 0.2043 | 0.75 | 0.0859 | 0.98 | 0.0334 | 1.00 | 0.07 | 0.79 |
|  |  | |  |  |  |  |  |  |  |  |  |  |
| *Hoxa5^+/+^* | 18, F | | 32.32 |  | 0.1318 |  | 0.0681 |  | 0.0226 |  | 0.0557 |  |
| *Hoxa5^-/-^* |  | | 26.9 | 0.83 | 0.0957 | 0.87 | 0.0316 | 0.56 | 0.0232 | 1.23 | 0.0573 | 1.24 |
|  |  | |  |  |  |  |  |  |  |  |  |  |
|  |  | |  |  |  |  |  |  |  |  |  |  |
|  |  | Average | | 0.99 |  | 0.85 |  | 0.77 |  | 1.04 |  | 1.05 |
|  | | Standard Deviation | | 0.13 |  | 0.09 |  | 0.18 |  | 0.31 |  | 0.19 |
